# Identification of shared genetic variants between schizophrenia and lung cancer

**DOI:** 10.1038/s41598-017-16481-4

**Published:** 2018-01-12

**Authors:** Verena Zuber, Erik G. Jönsson, Oleksandr Frei, Aree Witoelar, Wesley K. Thompson, Andrew J. Schork, Francesco Bettella, Yunpeng Wang, Srdjan Djurovic, Olav B. Smeland, Ingrid Dieset, Ayman H. Fanous, Rahul S. Desikan, Sébastien Küry, Stéphane Bézieau, Anders M. Dale, Ian G. Mills, Ole A. Andreassen

**Affiliations:** 1 NORMENT, KG Jebsen Centre for Psychosis Research, Institute of Clinical Medicine, University of Oslo, Oslo, Norway; 2 Division of Mental Health and Addiction, Oslo University Hospital, Oslo, Norway; 3 Centre for Molecular Medicine Norway, Nordic EMBL Partnership, University of Oslo and Oslo University Hospital, Oslo, Norway; 4 MRC Biostatistics Unit, University of Cambridge, Cambridge, UK; 5 Department of Clinical Neuroscience, Centre for Psychiatry Research, Karolinska Institutet, Stockholm, Sweden; 6 Department of Psychiatry, University of California, San Diego, La Jolla, CA USA; 7 Multimodal Imaging Laboratory, University of California at San Diego, La Jolla, CA USA; 8 Cognitive Sciences Graduate Program, University of California, San Diego, La Jolla, CA USA; 9 Center for Human Development, University of California at San Diego, La Jolla, CA USA; 10 NORMENT, KG Jebsen Centre for Psychosis Research, Department of Clinical Science, University of Bergen, Bergen, Norway; 11 Department of Medical Genetics, Oslo University Hospital, Oslo, Norway; 12 Department of Psychiatry, SUNY Downstate Medical Center, Brooklyn, NY USA; 13 Department of Radiology, University of California, San Francisco, San Francisco, CA USA; 14 CHU Nantes, Service de Génétique Médicale, 9 quai Moncousu, 44093, Nantes, CEDEX 1 France; 15 Department of Neurosciences, University of California, San Diego, La Jolla, CA USA; 16 Department of Cancer Prevention, Institute of Cancer Research and Department of Urology, Oslo University Hospital, Oslo, Norway; 17 Prostate Cancer UK/Movember Centre of Excellence for Prostate Cancer Research, Centre for Cancer Research and Cell Biology, Queen’s University Belfast, Belfast, BT9 7AE UK; 18 Nuffield Department of Surgical Sciences, University of Oxford, Oxford, United Kingdom

## Abstract

Epidemiology studies suggest associations between schizophrenia and cancer. However, the underlying genetic mechanisms are not well understood, and difficult to identify from epidemiological data. We investigated if there is a shared genetic architecture between schizophrenia and cancer, with the aim to identify specific overlapping genetic loci. First, we performed genome-wide enrichment analysis and second, we analyzed specific loci jointly associated with schizophrenia and cancer by the conjunction false discovery rate. We analyzed the largest genome-wide association studies of schizophrenia and lung, breast, prostate, ovary, and colon-rectum cancer including more than 220,000 subjects, and included genetic association with smoking behavior. Polygenic enrichment of associations with lung cancer was observed in schizophrenia, and weak enrichment for the remaining cancer sites. After excluding the major histocompatibility complex region, we identified three independent loci jointly associated with schizophrenia and lung cancer. The strongest association included nicotinic acetylcholine receptors and is an established pleiotropic locus shared between lung cancer and smoking. The two other loci were independent of genetic association with smoking. Functional analysis identified downstream pleiotropic effects on epigenetics and gene-expression in lung and brain tissue. These findings suggest that genetic factors may explain partly the observed epidemiological association of lung cancer and schizophrenia.

## Introduction

Schizophrenia (SCZ) is a mental disorder that greatly impacts the life of the affected individuals and ranks globally among the leading causes of disability. Genetic factors are important for development of SCZ, and heritability estimates range up to 0.8^1^. Large genome-wide association studies (GWAS) suggest that SCZ is a polygenic disease with many genetic variants associated, each with a small effect^2^. Recently, several lines of evidence indicate genetic overlap between SCZ and other brain disorders^3^ as well as cardiovascular risk factors^4^. Due to the polygenic nature of SCZ, it is possible that shared genetic factors may also underlie other diseases or traits associated with SCZ.

Epidemiological studies report both inverse and direct co-morbidity between SCZ and some cancer types. For example, a meta-analysis of cancer incidence in more than 500,000 participants showed an increased risk for breast cancer and decreased risk for melanoma and lung cancer^5^. Similarly, a prospective cohort study found increased risk of breast cancer for women and lung cancer for men^6^. Additional support for comorbidity between SCZ and lung cancer was given by a Danish nation-wide registry study^7^. In contrast, a large UK cohort study did not show any significant difference in incidence of colorectal cancer, breast cancer and lung cancer between SCZ and controls^8^. Another study investigating parents of patients with SCZ did not find any significantly reduced risk for overall cancer types, although it reported an increased risk for lung cancer in mothers of patients with SCZ^9^. Furthermore, first-degree relatives of patients with SCZ showed significantly reduced overall cancer risk^10^. In summary, the literature seems to provide inconsistent results. This can be due to study design, as well as confounders including lifestyle factors, such as smoking or diet, antipsychotic medication, and different approaches to cancer screening and treatment. Additionally, cancer is a disease of the older ages, while patients with SCZ have a decreased life expectancy of 10–25 years.

Combining GWAS from multiple disorders provides insights into genetic pleiotropy, a single genetic variant associated with more than one distinct phenotype, and could elucidate shared pathophysiology. We used a genetic epidemiology framework based on the conjunction false discovery rate (FDR), which enables identification of specific loci of cross-phenotype association independent of direction, thus making it particularly useful to test overlap between different diseases where directions of effects are unknown^4^. Since the FDR framework requires only summary statistics we were able to integrate GWAS data from SCZ and cancer sites from more than 220,000 subjects (Supplementary Table 1). Our first aim was to visualize polygenic overlap between SCZ and cancer in a genome-wide enrichment analysis and if this varies depending on cancer sites. Secondly, we aimed at identifying specific loci sharing association between SCZ and cancer using conjunction FDR, a two-dimensional extension of the FDR. Finally, we functionally characterized the shared loci using epigenetic and expression data in relevant tissue types to better understand joint disease etiologies.

## Results

### Enrichment pattern between schizophrenia (SCZ) and cancer

**Figure 1:**
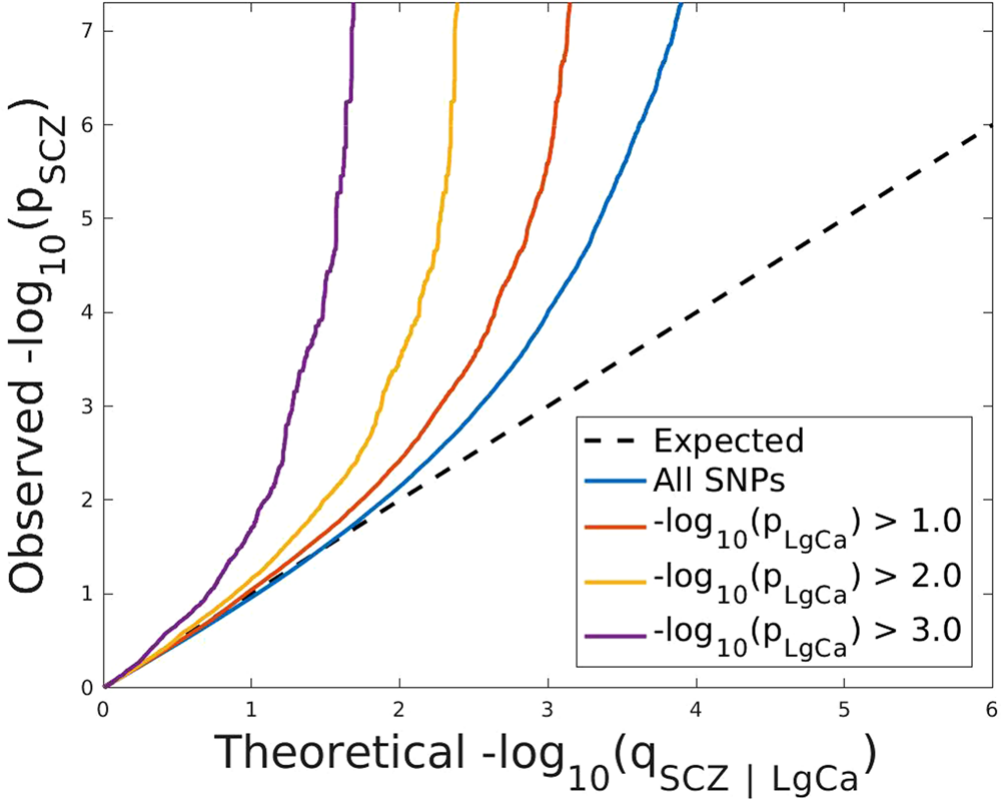
Stratified Q-Q plot for schizophrenia (SCZ) given lung cancer (LgCa). Stratified Q-Q plot of theoretical vs empirical −log_10_ *p*-values (corrected for genomic control) in schizophrenia (SCZ) below the standard GWAS threshold of -log10 *p*-values equal to 7.3 (equals *p*-values above 5 × 10^−8^) as a function of significance of association with lung cancer (LgCa) at the level of *p* < *1* (all SNPs), *p* < *0.1, p* < *0.01, p* < *0.001* respectively. Dotted lines indicate the theoretical line in case of no association. Prior to this analysis single nucleotide polymorphisms (SNPs) in the major histocompatibility complex (MHC) have been excluded.

A stratified quantile-quantile (Q-Q) plot showed a strong enrichment pattern for SCZ given lung cancer (Fig. 1). While the blue line shows the standard enrichment of the main trait of interest (SCZ) including all SNPs irrespective of their association with the secondary trait (lung cancer), we observe a stronger leftward deflection from the dashed line of no association with increasingly stronger association with lung cancer. We did not see any similar enrichment pattern for any other cancer sites. Breast cancer showed weak enrichment (Supplementary Figure 1A), i.e. strata conditional on association with breast cancer did not diverge from the line of all SNPs. Conditioning on prostate cancer did not result in any deflection (Supplementary Figure 1B) from the Q-Q line of all SNPs. Furthermore, there was no substantial enrichment given strata defined by ovarian cancer (Supplementary Figure 1C) or colon cancer (Supplementary Figure 1D), which might be due to the comparatively small sample sizes of these GWAS.

To test for statistical significance of enrichment for the Q-Q plot strata we used LD-score regression^11^. After adjusting for multiple testing (four cancer traits and three strata) we detected an increase in the enrichment parameter for SCZ given lung cancer ranging from 1.424 (−log10pval >1) to 2.190 (−log10pval >2), and 6.512 (-log10pval >3) of which the first stratum is significantly enriched after multiple testing correction, and the second stratum is nominally significant (Supplementary Table 2). None of the other traits showed significant enrichment of any strata. The prostate cancer study was excluded from the enrichment analysis since its coverage (211,155 SNPs) using a customised genotyping platform was too low. All analysis was performed after excluding SNPs mapping to the major histocompatibility complex (MHC, genomic position (hg 19): chr6:29,528,318- 33,373,649^12^) since the MHC has been shown to be one of the key driving factors for enrichment of genetic association in SCZ^13^. In order to check involvement of the MHC region, we repeated the stratified Q-Q plot for SCZ given lung cancer (Supplementary Figure 2) including all SNPs mapping to the MHC, but we did not find substantial changes in enrichment as seen in the stratified Q-Q plots between analysis including the MHC (Supplementary Figure 2) and excluding the MHC (Fig. 1). Further we note the symmetry of the observed enrichment and show the stratified Q-Q plot for lung cancer given SCZ in Supplementary Figure 3.

### Shared risk loci between schizophrenia (SCZ) and lung cancer

**Figure 2:**
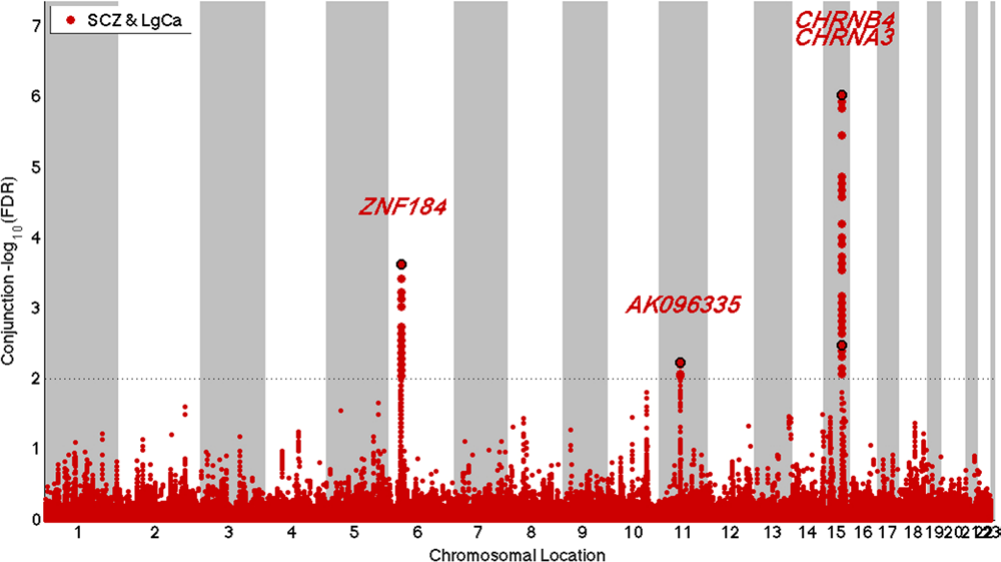
Manhattan plot for independent (*r* ^*2*^ *< 0.2*) loci associated with both schizophrenia (SCZ) and lung cancer (LgCa) as defined by conjunction false discovery rates (ConjFDR) < 0.01 after excluding single nucleotide polymorphisms in the major histocompatibility complex.

Three independent (*r* ^*2*^ *< 0.2*) loci shared between SCZ and lung cancer passed the conjunctional FDR < 0.01 threshold. See Table 1 for *p*-values and effect directions and Fig. 2 for the conjunctional FDR Manhattan plot. Variants mapping to the MHC have been removed prior to fitting the conjunction FDR.

**Table 1:** Independent (*r* ^2^ < *0.2*) loci associated with both schizophrenia (SCZ) and lung cancer (LgCa) as defined by conjunction false discovery rates (ConjFDR < 0.01).

SNP	Gene	Band	A1	A2	*p-*value	*p-*value	*p-*value	*z-*score	*z-s*core	*z*-score	ConjFDR	ConjFDR
					SCZ	LgCa	CPD	SCZ	LgCa	CPD	SCZ_LgCa	SCZ_CPD
rs7749305	ZNF184	6p22.1	T	C	2.385e-17	5.084e-06	NaN	8.47	−4.56	NaN	2.340e-04	NaN
rs2081361	AK096335	11q12.1	C	T	2.525e-04	1.667e-05	2.517e-01	−3.66	−4.31	−1.15	5.891e-03	1
rs8042374	CHRNA3	15q25.1	A	G	2.056e-08	5.302e-32	5.067e-23	5.61	11.77	9.88	9.317e-07	1.960e-05

In addition, we include cross-phenotype association of SCZ and smoking status (measured by number of cigarettes per day (CPD)). For each locus we report the lead single nucleotide polymorphism (SNP), closest annotated gene (Gene), genomic position (Band), *p*-values and *z*-scores with A1 (reference allele) and A2 (effect allele) for the specific traits. The major histocompatibility complex (MHC) was excluded from the analyses. The SNP rs7749305 on band 6p22.1 has the genomic position (hg19) chr6:27,446,566 and is thus outside the physical boundaries of the MHC. Still, it is an eQTL with a MHC-related gene (*BTN3A2*, Supplementary Table 3B). Not available number (NaN) if not available in the summary data file.

The three loci with joint association between SCZ and lung cancer were explored with functional follow-up studies. The strongest association was found for the locus on 15q25.1 mapping to genes of the nicotinic acetylcholine receptors, which has been previously implicated for cross-phenotype association between lung cancer and smoking^14^. The 15q25.1 locus showed a concordant effect direction between SCZ and lung cancer. There was one LD proxy (rs2904130, *r* ^*2*^ *= 0.89*) of the lead SNP rs8042374, which is an expression quantitative trait loci (eQTL) with neuronal acetylcholine receptor subunit alpha-5 (*CHRNA5)* and in both in lung and brain (caudate) tissue (Genotype-Tissue Expression (GTEx)^15^ Supplementary Table 3A). The locus on 6p22.1, has been identified and replicated as a cross-phenotype association between lung cancer and blood triglycerides^16^. This locus harbors two SNPs (rs28360634 and rs72839477) in strong LD (*r* ^*2*^ *= 1*) with the lead SNP rs7749305, which are eQTL (GTEx^15^) in both brain and lung tissue with the same gene butyrophilin subfamily 3 member A2 (*BTN3A2)*. The eQTL in brain tissue was confirmed in the independent Brain eQTL dataset (Braineac^17^, Supplementary Table 3B). The lead SNP rs7749305 is outside of the physical boundaries of the MHC, but it is an eQTL with *BTN3A2*, a MHC-related gene, underscoring the complicated and extensive LD structure in this region. The third association was on 11q12.1 and included the lead SNP rs2081361, which was an eQTL (GTEx) in lung tissue with the gene translocase of inner mitochondrial membrane 10 homolog (*TIMM10)* and with the leucine-rich repeat-containing protein 55 (*LRRCP55*) which is an auxiliary protein of the large-conductance, voltage and calcium-activated potassium channel. Further we found evidence for rs2081361 to be a moderate eQTL with *TIMM10* in brain tissue in the Braineac database (Supplementary Table 3B). We found epigenetic evidence for rs2081361 in lung tissue (normal human lung fibroblast (NHLF) and adenocarcinomic human alveolar basal epithelial cells (A549)), and Henrietta Lacks (HeLa) S3 cells (Supplementary Figure 4). In particular, for A549 and HeLa S3 we found CCCTC-binding factor (*CTCF*) binding and for NHLF open chromatin as characterized by DNase1 was discovered. A summary of the eQTL data is given in Supplementary Table 3 (A. GTEx^15^, B. Braineac^17^).

### Genetic overlap and shared risk loci between schizophrenia (SCZ) and smoking

Smoking is the main risk factor for lung cancer, and there is a higher prevalence of smoking among patients with SCZ than controls. There is also one reported cross-phenotype association between lung cancer and smoking^14,18^. Thus, we investigated if association with smoking behavior measured by cigarettes per day (CPD), correlated with the polygenic overlap between SCZ and lung cancer. As shown in the stratified Q-Q plot (Supplementary Figure 5A.), there is an enrichment of SCZ association given CPD (after removing MHC region). After removing SNPs mapping to the nicotinic acetylcholine receptors (genomic position (hg 19) chr15: 78,686,690-79,231,478) the enrichment of SCZ given CPD disappears (Supplementary Figure 5B) which suggests that the shared signal between SCZ and CPD is driven by genetic variation within the nicotinic acetylcholine receptors.

To detect cross-phenotype association between SCZ and smoking behavior we computed the conjunction FDR for joint association between SCZ and CPD. There is only one locus, 15q25.1, with conjunction FDR < 0.01 between SCZ and CPD. This has a concordant association between lung cancer and smoking, as reported earlier^14,18^, and the effect direction is also concordant for SCZ. Remarkably, the other two loci shared by SCZ and lung cancer had a conjunction FDR for SCZ and CPD close to one, which indicates no association between SCZ and CPD apart from the locus on 15q25.1 (Table 1). We included further smoking traits such as onset, cessation, and initiation into the analysis, but except for the locus on 15q25.1 none of the cross-phenotype associated SNPs shows any association with any other smoking trait (Supplementary Table 4).

### Shared risk loci between schizophrenia (SCZ) and squamous cell carcinoma type of lung cancer

Furthermore, we refined the definition of shared genetic variants between SCZ and lung cancer to subtypes of lung cancer, adenocarcinoma (ADENO) and squamous cell carcinoma (SQUAM). We analyzed the two subtypes and presented stratified Q-Q plots for SCZ given SQUAM (Supplementary Figure 6A) and for SCZ given ADENO (Supplementary Figure 6B). Noteworthy, we observed a strong enrichment for SCZ for SQUAM, and a weaker enrichment for ADENO. This is in line with previous findings of a different genetic architecture of the two cancer sub-types^19^.

We found three independent loci with conjunction FDR < 0.01 for SCZ&SQUAM, and one locus with conjunction FDR < 0.01 for SCZ&ADENO (Supplementary Table 5). The locus shared between SCZ, SQUAM and ADENO is the locus on 15q25.1, which was the strongest association in the general lung cancer analysis.

## Discussion

We report polygenic enrichment between SCZ and lung cancer, but not for any other cancer site. This suggests that shared genetic risk factors may underlie the association between SCZ and lung cancer shown in epidemiological studies. Smoking is strongly associated with both SCZ and lung cancer, and here we show that variants mapping to the nicotinic acetylcholine receptors may contribute to this overlap. The current findings of shared variants associated with these three phenotypes have implications for the underlying pathophysiological processes, and interpretation of epidemiological findings. In particular, the finding of partly genetic causes for the high smoking prevalence in SCZ are of clinical relevance. It underscores the importance of preventive measures against smoking initiation and smoking cessation programs in mental health care, and suggests evaluation of lung cancer screening programs in SCZ.

The conjunction FDR is a genome-wide approach and it is possible that inclusion of larger LD blocks such as the MHC can impact the model fit and confound the results. Therefore, the main results are based on the analysis after excluding the MHC and re-fitting the FDR estimate, which showed associations of three loci (6p22.1, 11q22.1, and 15q25.1). The statistical framework we used has the advantage of pinpointing loci of cross-phenotype associations even when the effect directions are mixed as it is the case for the three loci we identified here (Table 1). In contrast, LD score regression^11^, a useful approach for genome-wide co-heritability analysis as presented in Supplementary Table 2, is neither able to identify specific genetic regions nor pleiotropic traits with mixed effect direction^20^.

The locus on chromosome 15q25.1, including the nicotinic acetylcholine receptors *CHRNA3*, *CHRNA5* and *CHRNB4*, showed concordant effect direction between SCZ, lung cancer, and smoking behavior. When the two lung cancer sub-types were analyzed, the associations with SCZ were in same direction. The locus on chromosome 11q12.1 showed concordant effect direction for SCZ and lung cancer. It harbors several variants that are moderate eQTL in both lung and brain tissue with the gene translocate of inner mitochondrial membrane 10 (*TIMM10)*. The protein encoded by *TIMM10* functions as a preprotein translocase for the import of proteins into inner and outer membranes, particularly inner membrane metabolite carriers^21^. Under-expression of genes of the TIMM family has been associated with neurodegenerative diseases^22^. Additionally *TIMM10* transcript was recently identified as significantly down-regulated in dorsolateral prefrontal cortex layer 3 pyramidal cells isolated from tissue from SCZ patients^23^. However, the present evidence for involvement of *TIMM10* is moderate and replication and further investigations are needed.

We found associations between SCZ and both histological types of lung cancer, squamous cell carcinoma and adenocarcinoma. The enrichment was stronger and more extensive in the squamous cell type, which had three loci associated with SCZ, and only one with adenocarcinoma (the *CHRNA3/CHRNA5/CHRNB4* cluster on chromosome 15q25.1). It was reported that more than 90% of patients with squamous cell carcinoma were or had been smokers, as compared to about 55% of those suffering from adenocarcinoma^24^.

We expect that the present findings will form the basis for future studies of the role of the 15q25.1 in smoking behavior, lung cancer, and SCZ. Our approach aimed at identifying cross-phenotype associations and cannot distinguish between biological and mediated pleiotropy^25^. The present findings demonstrate the importance of further functional follow-up studies and further investigations using other approaches such as Mendelian Randomisation, which can help to distinguish between biological and mediated pleiotropy. Recent epidemiological studies, including a Mendelian Randomisation study^26^ and a prospective co-relative control study^27^ have found evidence for smoking initiation as putative risk factor for SCZ.

The present findings suggest pleiotropic downstream effects of the cross-phenotype associations. Especially eQTL studies in relevant tissue types provide important insights how genetic variants exert downstream effects on gene-expression^28^. The evidence that all three cross-phenotype associations from our pleiotropic analysis are eQTL with the same gene (nicotinic acetylcholine receptors, *BTN3A2*, *TIMM10*) in relevant tissue types including lung and brain further support the claim of downstream pleiotropy between SCZ and lung cancer and complement observed associations from epidemiology studies. Further analyses of the molecular downstream consequences of these genetic variants are beyond the scope of this manuscript. One should be cautious with interpretation, as the relationship between SCZ and lung cancer is complex. Cancer risk in SCZ seems to vary with age, with higher than expected frequencies during young ages and lower than expected frequencies later in life^29^. Also lung cancer followed this pattern, with higher standardized incidence ratios at ages less than 60 years, and lower incidences at higher ages^29^. We do not have data stratified for age in the present study.

In conclusion, we identified shared genetic variation between SCZ and lung cancer in the *CHRNA3/CHRNA5/CHRNB4* cluster on chromosome 15q25.1, and two other loci (6p22.1, 11q12.1) show cross-phenotype association and downstream pleiotropic effects on gene-expression in relevant tissue types for lung cancer and SCZ. The genetic effects are however complex, giving rise to both increased and decreased risk of the disorders. Further efforts into fine-mapping, causal analysis, and functional annotation are needed to clarify how these cross-phenotype associations exert their pleiotropic effects. Especially of interest is the role of the nicotinic acetylcholine receptors in the synthesis of smoking behavior, lung cancer and SCZ.

## Methods

### Genome-wide association studies (GWAS) Samples

GWAS summary statistics on SCZ were provided by the Psychiatric Genomic Consortium (PGC) and comprised association analyses of 32,405 cases and 42,221 controls^2^. The summary statistics on five cancer sites were obtained from the Genetic Associations and Mechanisms in Oncology (GAME-ON) consortium and included lung cancer (13,373 cases and 26,014 controls)^19^ (including sub-types referred to as adenocarcinoma (ADENO) and squamous cell carcinoma (SQUAM)), breast cancer (15,863 cases and 40,022 controls)^30^, prostate cancer (25,074 cases and 24,272 controls)^31^, colon cancer (5,100 cases and 7,529 controls)^32^, and ovarian cancer (3,995 cases and 3,277 controls)^33^. Additionally, we included GWAS data on smoking behavior measured by cigarettes per day (CPD) (74,503 individuals)^34^. For more details see Supplementary Table 1.

### Pre-processing

As a first pre-processing step we aligned all summary statistics to a common set of reference single nucleotide polymorphisms (SNPs) (of size *d* = *2,558,411*) generated from the 1000 genomes project. As summary statistics we saved for each reference SNP and each trait one *p-*value and one *z-*score. Next we performed genomic control^35^, and finally we adjusted for overlap between samples^36^. There were overlaps between controls of the PGC study on SCZ and controls of the cancer studies, i.e. *n* = *3,179* individuals for lung cancer, *n* = *4,834* for breast cancer, and *n* = 713 for colon cancer. All *p*-values reported are adjusted for genomic control, all false discovery rates reported are adjusted for genomic control and sample overlap. As reference panel for the computation of the linkage disequilibrium (LD) structure between SNPs we use the European populations from the 1000 genomes project. The European population best reflects the mainly European composition of the PGC study on SCZ and the lung cancer GWAS.

### Quantile-Quantile (Q-Q) plots

Q-Q plots are standard tools in genomics to visualize the distribution of the observed *p-values* with the expected distribution of *p*-values under the null hypothesis, or in other words under no association of the tagged SNPs with the phenotype of interest. Q-Q plots depict the quantiles of the observed *p*-values on the y-axis against the theoretical quantiles under no association on the x-axis. In order to focus on the tails, Q-Q plots are often displayed on the −*log10* scale. In case of no association a Q-Q plot follows a straight line. Deflection from this null line describes enrichment, i.e. the presence of lower *p*-values as expected by chance. Stratified Q-Q plots investigate differential enrichment between pre-specified strata of SNPs^37,38^. When investigating polygenic shared architecture between two traits we focused on the *p*-values of trait 1 (SCZ), and defined the strata based on trait 2 (cancer). More specifically we plotted the *p*-values of trait 1 given or conditional on different strength of association with trait 2 (i.e. *p*-value > *−log10 p*-values of 1, 2, or 3). Thus, we were able to visualize if conditioning on a secondary trait leads to stronger enrichment in the primary trait of interest. A strong enrichment increasing with association on the secondary trait is an indicator of a shared polygenic architecture between the two traits.

Large blocks of linkage disequilibrium (LD) may confound the results. To account for this we applied a random pruning approach, where one random SNP per LD block (defined by an *r* ^2^ of 0.8) were used and averaged over 100 random pruning runs. The impact of differing correlation parameters (from 0.7 to 0.3) on the Q-Q plots is displayed in Supplementary Figure 7. Further we focus the Q-Q plots on the region below genome-wide significance (*−log10 p-*values < 7.3) in order to highlight the polygenic component of the cross-phenotype association.

In order to test for differential enrichment of the Q-Q plot strata we use LD score regression^11^ to test for fold enrichment. We assess the fold enrichment of each of the three strata (i.e. *p*-value > *−log10 p*-values of 1, 2, or 3) represented in the stratified Q-Q plots with the total LD score as covariate. The prostate cancer study was excluded from the analysis since its coverage (211,155 SNPs) using a customised genotyping platform was too low. Multiple-testing correction is performed for four cancer traits and for the three strata (*p*_adjusted = *p*–value × 4 cancer types × 3 strata).

### Conditional and conjunction false discovery rate (FDR)

The second part of our genetic epidemiology framework aimed at pinpointing shared cross-phenotype associations using the conjunction false discovery rate (FDR). The basic FDR framework is based on the assumption that the distribution of *p-*values follows a mixture distribution where SNPs are either associated (non-null) or not associated (null) with the phenotype^39^. The (tail-area based) FDR is defined as the probability that a given SNP is null given that its *p-*value is as small as or smaller than the observed one. Note that in context of the FDR all modeling is done on the summary statistic level, and no access to genotype data is needed. The conditional FDR is a simple extension of the standard FDR that allows including additional information on the association of a SNP with a secondary trait or more precisely, with the *p*-value of the same SNP in a secondary trait. It is defined as the probability that a specific SNP is null given that the *p*-values for both, trait 1 and trait 2, are as small as or smaller than the observed ones^37,38^. Low values of conditional FDR can be driven by the first trait only. To detect SNPs associated jointly with both traits at the same time we employed the conjunction FDR. It is defined as the probability of being null for either trait, or for both traits simultaneously given that the *p*-values for the two traits are as small as or smaller than the observed ones. Thus, a true discovery is only the case when a SNP is non-null for both traits jointly. This symmetric behavior of the conjunction FDR weights both traits equal. Low values in conjunction FDR can only be found when a SNP is associated with both traits jointly. For example, for lung cancer and SCZ this symmetric behavior is best demonstrated by a stratified Q-Q plot of SCZ given lung cancer and then vice-versa lung cancer given SCZ (Supplementary Figure 3).

For more information on conditional and conjunction FDR we refer to^40^. We set a conservative FDR level of 0.01 per pair-wise comparison, which relates to one expected false positive finding within 100 reported findings. The conjunction FDR provides a genome-wide unbiased scan and is thus a suitable technique to discover novel associations that are not detected by a univariate conservative Bonferroni threshold.

### Functional follow up

To investigate downstream effects of the cross-phenotype associated genetic loci we looked up expression quantitative trait loci (eQTL) in relevant tissue types (especially lung and brain) in the Genotype-Tissue Expression (GTEx) database^15^, and the UK Brain Expression Consortium (Braineac)^17^.

## Electronic supplementary material

Supplementary Information
