# Supplementary material for: Identification of shared genetic variants between schizophrenia and lung cancer
Source: Sci Rep. 2018 Jan 12;8:674. doi: 10.1038/s41598-017-16481-4 (PMC5766533; doi:10.1038/s41598-017-16481-4)
Supplement: Supplementary file 1 — Supplementary Information [file 41598_2017_16481_MOESM1_ESM.doc]

**Identification of shared genetic variants between schizophrenia and lung cancer -Supplementary Material**

Verena Zuber^1,2,3,4^, Erik G. Jönsson^1,5^, Oleksandr Frei^1,2^, Aree Witoelar^1,2^, Wesley K. Thompson^6^, Andrew J. Schork^7,8,9^, Francesco Bettella^1,2^, Yunpeng Wang^1,2^, Srdjan Djurovic^2,10^, Olav B. Smeland^1,2^, Ingrid Dieset^1,2^, Ayman H. Fanous^11^, Rahul S. Desikan^12^, Sébastien Küry^13^, Stéphane Bézieau^13^, Colon CFR^a^, ColoRectal Transdisciplinary Study (CORECT)^b^, Discovery, Biology, and Risk of Inherited Variants in Breast Cancer (DRIVE), Follow-up of Ovarian Cancer Genetic Association and Interaction Studies (FOCI)^c^, the PRACTICAL Consortium^d^, Transdisciplinary Research in Cancer of the Lung (TRICL) Research Team^e^, Anders M. Dale^6,7,9,14^, Ian G. Mills^3,15,16^, Ole A. Andreassen^1,2,§^

^1^NORMENT, KG Jebsen Centre for Psychosis Research, Institute of Clinical Medicine, University of Oslo, Oslo, Norway

^2^Division of Mental Health and Addiction, Oslo University Hospital, Oslo, Norway

^3^Centre for Molecular Medicine Norway, Nordic EMBL Partnership, University of Oslo and Oslo University Hospital, Oslo, Norway

^4^MRC Biostatistics Unit, University of Cambridge, Cambridge, UK.

^5^Department of Clinical Neuroscience, Centre for Psychiatry Research, Karolinska Institutet, Stockholm, Sweden

^6^Department of Psychiatry, University of California, San Diego, La Jolla, CA, USA

^7^Multimodal Imaging Laboratory, University of California at San Diego, La Jolla, CA, USA

^8^Cognitive Sciences Graduate Program, University of California, San Diego, La Jolla, CA, USA

^9^Center for Human Development, University of California at San Diego, La Jolla, CA, USA

^10^ NORMENT, KG Jebsen Centre for Psychosis Research, Department of Clinical Science, University of Bergen, Bergen, Norway

^11^Department of Psychiatry, SUNY Downstate Medical Center, Brooklyn NY, USA

^12^Department of Radiology, University of California, San Francisco, San Francisco, CA, USA

^13^CHU Nantes, Service de Génétique Médicale, 9 quai Moncousu, 44093, Nantes CEDEX 1, France

^14^Department of Neurosciences, University of California, San Diego, La Jolla, CA, USA

^15^Department of Cancer Prevention, Institute of Cancer Research and Department of Urology, Oslo University Hospital, Oslo, Norway

^16^ Prostate Cancer UK/Movember Centre of Excellence for Prostate Cancer Research, Centre for Cancer Research and Cell Biology, Queen’s University Belfast, BT9 7AE, UK

^a^Members of the The Colon Cancer Family Registries (Colon CFR) are provided in the Supplementary information.

^b^Members of the ColoRectal Transdisciplinary Study (CORECT) are provided in the Supplementary information.

^c^Members of the Follow-up of Ovarian Cancer Genetic Association and Interaction Studies (FOCI) Research Team are provided in the Supplementary information.

^d^Members from the Prostate Cancer Association Group to Investigate Cancer Associated Alterations in the Genome (PRACTICAL) consortium are provided in the Supplementary information. Information of the consortium can be found at <http://practical.ccge.medschl.cam.ac.uk/>.

^e^Members of the Transdisciplinary Research in Cancer of the Lung (TRICL) Research Team are provided in the Supplementary information.

.

This work would not have been possible without the ColoRectal Transdisciplinary Study (CORECT) consortium, the Discovery, Biology, and Risk of Inherited Variants in Breast Cancer (DRIVE) consortium, the Follow-up of Ovarian Cancer Genetic Association and Interaction Studies (FOCI) Research Team, the Prostate Cancer Association Group to Investigate Cancer Associated Alterations in the Genome (PRACTICAL) Consortium, Transdisciplinary Research in Cancer of the Lung (TRICL) Research Team. Here is a detailed list of participants and members for each consortium.

**The Colon Cancer Family Registries (Colon CFR) Authors**

Dennis Ahnen^16^, Kristen Anton^17^, John Baron^18^, Daniel D. Buchanan^19^, Graham Casey^20^, James Church^21^, Tim Church^22^, Michelle Cotterchio^23^, Jane C. Figueiredo^20^, Steven J. Gallinger^24^, Robert W. Haile^25^, John L. Hopper^26^, Mark A. Jenkins^26^, Peter Lance^27^, Loic Le Marchand^28^, Noralane M. Lindor^29^, Polly A. Newcomb^30^, Stephen N. Thibodeau^31^

^16^Division of Gastroenterology, School of Medicine, university of Colorado, Aurora, CO 80045, USA. ^17^Department of Biomedical Data Science, Geisel School of Medicine, Dartmouth College, Lebanon, NH 03766, USA. ^18^Division of Gastroenterology and Hepatology, Department of Medicine, University of North Carolina, Chapel Hill, NC 27599, USA. ^19^Colorectal Oncogenomics Group, Department of Pathology, University of Melbourne, Parkville, Victoria 3010, Australia. ^20^Department of Preventive Medicine, USC Norris Comprehensive Cancer Center, Keck School of Medicine, University of Southern California, Los Angeles, California 90089, USA. ^21^Department of Colorectal Surgery, Cleveland Clinic Digestive Disease Institute. Cleveland Clinic, Cleveland, OH 44195, ^22^Division of Environmental Health Sciences, School of Public Health, University of Minnesota, Minneapolis MN 55455, USA. ^23^Dalla Lana School of Public Health, University of Toronto and Cancer Care Ontario, Toronto, Canada. ^24^Zane Cohen Centre for Digestive Diseases, Mount Sinai Hospital, Toronto, Ontario M5T 3L9, Canada. ^25^Department of Medicine, Division of Oncology, Stanford University, Stanford, CA 94305, USA. ^26^Centre for Epidemiology and Biostatistics, Melbourne School of Population and Global Health, The University of Melbourne, Parkville, Victoria 3010, Australia. ^27^Department of Medicine, The University of Arizona Cancer Center, University of Arizona, Phoenix, AZ , USA. ^28^Epidemiology Program, University of Hawaii Cancer Center, Honolulu, Hawaii 96822, USA. ^29^Department of Health Science Research, Mayo Clinic, Scottsdale, Arizona, 85259, USA. ^30^Public Health Sciences Division, Fred Hutchinson Cancer Research Center, Seattle, Washington 98124, USA. ^31^Department of Laboratory Medicine and Pathology, Mayo Clinic, Rochester, MN, 55905, USA.

**CORECT Authors**

Aaron K. Aragaki^32^, Sonja Berndt^33^, Hermann Brenner^34^, Daniel D. Buchanan^34^, Katja Butterbach^35^, Bette Caan^36^, Peter T. Campbell^37^, Christopher S. Carlson^32^, Graham Casey^38^, Jenny Chang-Claude^39^, Stephen J. Chanock^33^, Andrew T. Chan^40,41^, Gerhard A. Coetzee^42^, David V. Conti^38,43^, Keith R. Curtis^32^, David Duggan^44^, Christopher K. Edlund^38^, Jane C. Figueiredo^38^, Christopher P. Fischer^45^, Barbara K. Fortini^46^, Charles S. Fuchs^47,48^, Manish Gala^40^, Steven J. Gallinger^49^, W. James Gauderman^38^, Graham Giles^50^, Edward L. Giovannucci^42,48^, Stephanie Gogarten^51^, Roger Green^52^, Stephen B. Gruber^38,53^, Robert W. Haile^54^, John F. Harju^45^, Tabitha A. Harrison^32^, Richard B. Hayes^55^, Brian Henderson^38^, Michael Hoffmeister^34^, John L. Hopper^56^, Li Hsu^32^, Shu-Chen Huang, Thomas J. Hudson^57^, David J. Hunter^58^, Carolyn M. Hutter^59^, Gregory E. Idos^38^, Motoki Iwasaki^60^, Rebecca D. Jackson^61^, Eric Jacobs^38^, Sun Ha Jee^62^, Mark Jenkins^35^, Wei-Hua Jia^63^, Shuo Jiao^32^, Amit Joshi^64^, Laurence N. Kolonel^65^, Suminori Kono^66^, Charles Kooperberg^32^, Andrea LaCroix^32^, Cathy C. Laurie^51^, Loic Le Marchand^68^, Flavio Lejbkowicz^69,70^, Matieu Lemire^71^, David Levine^51^, Li Li^72^, Noralene M. Lindor^73^, Jing Ma^74^, Keren W. Makar^32^, Frank J. Manion^45^, Sanford D. Markowitz^75^, Keitaro Matsuo^66^, Kevin J. McDonnell^38^, Caroline E. McNeil^38^, Marilena Melas^38^, Victor Moreno^76^, Bhramar Mukherjee^45^, Polly A. Newcomb^32^, Kenneth Offit^77^, Ulrike Peters^32^, John D. Potter^32^, Conghui Qu^32^, Leon Raskin^78,79^, Gad Rennert^69,70,80^, Hedy Rennert^69,70^, Staephanie Rosse^32^, Stephanie L. Schmit^81^, Robert E. Schoen^82^, Fredrick R. Schumacher^83,84^, Daniela Seminara^85^, Gianluca Severi^86^, Wei Shi^87^, Xiao-Ou Shu^78,79^, Martha L. Slattery^88^, Darin Taverna^89^, Stephen N. Thibodeau^90^, Duncan C. Thomas^38^, Shoichiro Tsugane^30^, Cornelia M. Ulrich^91^, David J. Van Den Berg^38^ Hansong Wang^38^, Dee W. West^92^, Emily White^32^, Kana Wu^93^, Yong-Bing Xiang^94^, Brent W. Zanke^95,96^, Ben Zhang^97^, Wei Zheng^98,99^

^32^ Public Health Sciences Division, Fred Hutchinson Cancer Research Center, Seattle, Washington 98124, USA. ^33^Division of Cancer Epidemiology and Genetics, National Cancer Institute, Bethesda, Maryland 20892-9776, USA. ^34^Centre Hospitalier Universitaire Hotel-Dieu, Nantes 44093, France. ^35^Division of Clinical Epidemiology and Aging Research, German Cancer Research Center (DKFZ), Heidelberg 69121, Germany. ^36^Centre for Epidemiology and Biostatistics, Melbourne School of Population and Global Health, The University of Melbourne, Parkville, Victoria 3010, Australia. ^37^Division of Research, Kaiser Permanente Medical Care Program of Northern California, Oakland, California 94612, USA. ^38^Epidemiology Research Program, American Cancer Society, Atlanta, Georgia 30329-4251, USA. ^39^Department of Preventive Medicine, USC Norris Comprehensive Cancer Center, Keck School of Medicine, University of Southern California, Los Angeles, California 90089, USA. ^40^Unit of Genetic Epidemiology, Division of Cancer Epidemiology, German Cancer Research Center (DKFZ), Heidelberg 69121, Germany. ^41^Division of Gastroenterology, Massachusetts General Hospital, Boston, Massachusetts 02114, USA. ^42^Harvard Medical School, Boston, Massachusetts 02114, USA. ^43^Van Andel Research Institute, Grand Rapids, MI 49503 USA. ^44^Department of Pathology, Keck School of Medicine, University of Southern California, Los Angeles, California 90089, USA. ^45^Genetic Basis of Human Disease Division, Translational Genomics Research Institute, Phoenix, Arizona 85004, USA. ^46^University of Michigan Comprehensive Cancer Center, Ann Arbor, Michigan 48105, USA. ^47^Claremont Colleges, Claremont, CA 91711, USA. ^48^Department of Medicine, Brigham and Women’s Hospital, Brookline, Massachusetts 02115, USA. ^49^Department of Medical Oncology, Dana-Farber Cancer Institute, Brookline, Massachusetts 02115, USA. ^50^Zane Cohen Centre for Digestive Diseases, Mount Sinai Hospital, Toronto, Ontario M5T 3L9, Canada. ^51^ Cancer Epidemiology Centre, Cancer Council Victoria, Melbourne, Victoria 3004, Australia. ^52^Department of Biostatistics, University of Washington, Seattle, Washington 98195, USA. ^53^Discipline of Genetics, Memorial University of Newfoundland, St. John’s, Newfoundland A1B 3V6, Canada. ^54^Department of Medicine, Keck School of Medicine, University of Southern California, Los Angeles, California 90089, USA. ^55^Department of Medicine, Division of Oncology, Stanford University, Stanford, CA 94305, USA. ^56^Division of Epidemiology, Department of Population Health, New York University School of Medicine, New York, New York 10016, USA. ^57^Centre for MEGA Epidemiology, The University of Melbourne, Carlton, Victoria 3010, Australia. ^58^Department of Genomics, Ontario Institute for Cancer Research, Toronto, Ontario M5G 0A3, Canada. ^59^Program in Genetic Epidemiology and Statistical Genetics, Department of Epidemiology, Harvard School of Public Health, Boston, Massachusetts 02115, USA. ^60^Division of Cancer Control and Population Sciences, National Cancer Institute, National Institutes of Health, Rockville, Maryland 20892, USA. ^61^Research Center for Cancer Prevention and Screening, National Cancer Center, Tokyo 104-0045, Japan. ^62^Department of Medicine, Ohio State University, Columbus, Ohio 43210, USA. ^63^Department of Epidemiology and Health Promotion, Graduate School of Public Health, Yonsei University, Seoul 120-749, South Korea. ^64^State Key Laboratory of Oncology in South China, Cancer Center, Sun Yat-sen University, Guangzhou 510060, China. ^65^ Department of Epidemiology, Harvard School of Public Health, Boston, Massachusetts 02115, USA. ^66^ Office of Public Health Studies, University of Hawaii Manoa, Honolulu, Hawaii 96822, USA. ^67^Department of Preventive Medicine, Kyushu University, Fukuoka 812-8582, Japan. ^68^Service de Génétique Médicale, CHU Nantes, Nantes 44093, France. ^69^Epidemiology Program, University of Hawaii Cancer Center, Honolulu, Hawaii 96822, USA. ^70^Department of Community Medicine and Epidemiology, Carmel Medical Center, Haifa 34361, Israel. ^71^Clalit Health Services National Cancer Control Center, Haifa 34361, Israel. ^72^Ontario Institute for Cancer Research, Toronto, Ontario M5G 0A3, Canada. ^73^Department of Family Medicine and Community Health, Case Western Reserve University, Cleveland, Ohio 44106, USA. ^74^Department of Health Science Research, Mayo Clinic, Scottsdale, Arizona, 85259, USA. ^75^Harvard School of Public Health, Boston, Massachusetts 02114, USA. ^76^Case Western Reserve University, Case Comprehensive Cancer Center, Cleveland, Ohio 44106, USA. ^77^Catalan Institute of Oncology, IDIBELL and CIBERESP, Cancer Prevention and Control Program, Hospitalet, Barcelona, Spain. ^78^Memorial Sloan Kettering Cancer Center (MSKCC). ^79^Division of Epidemiology, Vanderbilt Epidemiology Center, Vanderbilt University School of Medicine, Nashville, Tennessee 37203-1738, USA. ^80^Vanderbilt-Ingram Cancer Center, Vanderbilt University, Nashville, Tennessee 37203-1738, USA. ^81^Bruce Rappaport Faculty of Medicine, Technion-Israel Institute of Technology, Haifa 3200003, Israel. ^82^Moffitt Cancer Center, Tampa, FL 33612, USA. ^83^Department of Internal Medicine, University of Pittsburgh Medical Center, Pittsburgh, Pennsylvania 15213, USA. ^84^Department of Epidemiology and Biostatistics, Case Western Reserve University, Cleveland, Ohio, 44106, USA. ^85^Seidman Cancer Center, University Hospitals, Cleveland, Ohio, 44106, USA.  ^86^Epidemiology and Genomics Research Program, Division of Cancer Control and Population Sciences, National Cancer Institute, National Institutes of Health, Bethesda, Maryland 20892, USA. ^87^Human Genetics Foundation (HuGeF), Torino 10126, Italy. ^88^ Department of Surgery, Children’s Hospital Los Angeles, Los Angeles, California 90027, USA. ^89^Department of Internal Medicine, University of Utah Health Sciences Center, Salt Lake City, Utah 84132, USA. ^90^Phoenix College, Phoenix, Arizona 85013, USA. ^91^Department of Laboratory Medicine and Pathology, Mayo Clinic, Rochester, MN, 55905, USA. ^92^Huntsman Cancer Institute, Salt Lake City, UT, 84112, USA. ^93^Cancer Registry of Greater California, Public Health Institute, Sacramento, California 95825, USA. ^94^Department of Nutrition, Harvard School of Public Health, Boston, Massachusetts 02115, USA. ^95^Department of Epidemiology, Shanghai Cancer Institute, Shanghai 2200-25, China. ^96^The University of Ottawa, Ottawa, Ontario K1N 6N5, Canada. ^97^Clinical Epidemiology Program, Ottawa Hospital Research Institute, Ottawa, Ontario, K1Y 4E9 Canada. ^98^Department of General Surgery, Third Military Medical University Southwest Hospital, Chongqing 400038, China.

**Follow-up of Ovarian Cancer Genetic Association and Interaction Studies (FOCI) Research Team**

Berchuck A^99^, Chen A^100^, Chenevix-Trench G^101^, Doherty J^102^, Fridley B^103^, Gayther S^104^, Goode E^105^, Iverson E^106^, Lawrenson K^107^, Lin H-Y^108^, Monteiro A^109^, Pharoah P^110,111^, Permuth JB^111^, Pearce L^112,113^, Phelan C^111^, Ramus S^112^, Risch H^114^, Reid B^115^, Rossing MA^116^, Schildkraut J^117^, Sellers TA^109^, Weber Palmieri R^118^, Wu A^112^

99. Department of Obstetrics and Gynecology, Duke University Medical Center, Durham, North Carolina, USA.

100. Department of Biostatistics and Bioinformatics, Moffitt Cancer Center, Tampa, FL, USA.

101. Genetics and Computational Biology Department, QIMR Berghofer Medical Research Institute, Queensland, AUS

102. Department of Epidemiology, Geisel School of Medicine, Dartmouth College, Hanover, NY, USA.

103. Department of Biostatistics, University of Kansas Medical Center, Kansas City, KS, USA.

104. Department of Biomedical Sciences, Samuel Oschin Comprehensive Cancer Institute, Cedars-Sinai Medical Center, Los Angeles, CA, USA

105. Department of Health Science Research, Division of Epidemiology, Mayo Clinic, Rochester, MN, USA.

106. Department of Statistical Science, Duke University, Durham, NC, USA.

107. Department of Obstetrics and Gynecology, Women's Cancer Program at the Samuel Oschin Comprehensive Cancer Institute, Cedars-Sinai Medical Center, Los Angeles, California.

108. School of Public Health, Louisiana State University Health Sciences Center, New Orleans, LA, USA

109. Department of Cancer Epidemiology, Moffitt Cancer Center, Tampa, FL, USA.

110. Department of Public Health and Primary Care, University of Cambridge, Strangeways Research Laboratory, Cambridge, UK.

111. Department of Oncology, University of Cambridge, Strangeways Research Laboratory, Cambridge, UK.

112. Department of Preventive Medicine, Keck School of Medicine, University of Southern California Norris Comprehensive Cancer Center, Los Angeles, California, USA.

113. Department of Epidemology,University of Michigan School of Public Health, Ann Arbor, MI, USA.

114. Department of Chronic Disease Epidemiology, Yale School of Public Health, New Haven, Connecticut, USA.

115. Department of Cancer Epidemiology, Division of Population Sciences, Moffitt Cancer Center, Tampa, FL, USA

116. Program in Epidemiology, Division of Public Health Sciences, Fred Hutchinson Cancer Research Center, Seattle, WA, USA.

117. School of Medicine, Public Health Sciences, University of Virginia, Charlottesville, VA.

118. Department of Community and Family Medicine, Duke University Medical Center, Durham, NC, USA.

**The PRACTICAL Consortium (http://practical.ccge.medschl.cam.ac.uk/):**

Rosalind Eeles ^119, 120^, Doug Easton ^121^, Zsofia Kote-Jarai ^119^, Ali Amin Al Olama ^121^, Sara Benlloch ^121^, Kenneth Muir ^122^, Graham G. Giles ^123, 124^, Fredrik Wiklund ^125^, Henrik Gronberg ^125^, Christopher A. Haiman ^126^, Johanna Schleutker ^127, 128^, Maren Weischer ^129^, Ruth C. Travis ^130^, David Neal ^131^, Paul Pharoah ^132^, Kay-Tee Khaw ^133^, Janet L. Stanford ^134, 135^, William J. Blot ^136^, Stephen Thibodeau ^137^, Christiane Maier ^138^, Adam S. Kibel ^139, 140^, Cezary Cybulski ^141^, Lisa Cannon-Albright ^142^, Hermann Brenner ^143,144,145^, Jong Park ^146^, Radka Kaneva ^147^, Jyotsna Batra ^148^, Manuel R. Teixeira ^149^, Hardev Pandha^150^, Yong-Jie Lu ^151^

^119^ The Institute of Cancer Research, London, SM2 5NG, UK,

^120^ Royal Marsden NHS Foundation Trust, London, SW3 6JJ, UK,

^121^ Centre for Cancer Genetic Epidemiology, Department of Public Health and Primary Care, University of Cambridge, Strangeways Research Laboratory, Worts Causeway, Cambridge, UK,

^122^ University of Warwick, Coventry, UK,

^123^ Cancer Epidemiology Centre, The Cancer Council Victoria, 615 St Kilda Road, Melbourne, Victoria, 3004, Australia,

^124^ Centre for Epidemiology and Biostatistics, Melbourne School of Population and Global Health, The University of Melbourne, Melbourne, Victoria, Australia,

^125^ Department of Medical Epidemiology and Biostatistics, Karolinska Institute, Stockholm, Sweden,

^126^ Department of Preventive Medicine, Keck School of Medicine, University of Southern California/Norris Comprehensive Cancer Center, Los Angeles, California, USA,

^127^ Department of Medical Biochemistry and Genetics, Institute of Biomedicine, Kiinamyllynkatu 10, FI-20014 University of Turku; and Tyks Microbiology and Genetics, Department of Medical Genetics, Turku University Hospital,

^128^ BioMediTech, 30014 University of Tampere, Tampere, Finland,

^129^ Department of Clinical Biochemistry, Herlev Hospital, Copenhagen University Hospital, Herlev Ringvej 75, DK-2730 Herlev, Denmark,

^130^ Cancer Epidemiology, Nuffield Department of Population Health, University of Oxford, Oxford, OX3 7LF, UK,

^131^ Nuffield Department of Surgery, University of Oxford, Room 6603, Level 6, John Radcliffe Hospital, Headley Way, Headington, Oxford, OX3 9DU, UK and University of Cambridge, Department of Oncology, Box 279, Addenbrooke's Hospital, Hills Road Cambridge CB2 0QQ, UK, ^132^ Centre for Cancer Genetic Epidemiology, Department of Oncology, University of Cambridge, Strangeways Research Laboratory, Worts Causeway, Cambridge, UK,

^133^ Cambridge Institute of Public Health, University of Cambridge, Forvie Site, Robinson Way, Cambridge CB2 0SR,

^134^ Division of Public Health Sciences, Fred Hutchinson Cancer Research Center, Seattle, Washington, USA,

^135^ Department of Epidemiology, School of Public Health, University of Washington, Seattle, Washington, USA,

^136^ International Epidemiology Institute, 1455 Research Blvd., Suite 550, Rockville, MD 20850,

^137^ Mayo Clinic, Rochester, Minnesota, USA,

^138^Institute of Human Genetics, University Hospital of Ulm, Ulm, Germany,

^139^ Brigham and Women's Hospital/Dana-Farber Cancer Institute, 45 Francis Street- ASB II-3, Boston, MA 02115,

^140^ Washington University, St Louis, Missouri,

^141^ International Hereditary Cancer Center, Department of Genetics and Pathology, Pomeranian Medical University, Szczecin, Poland,

^142^ Division of Genetic Epidemiology, Department of Medicine, University of Utah School of Medicine,

^143^ Division of Clinical Epidemiology and Aging Research, German Cancer Research Center (DKFZ), Heidelberg Germany,

^144^Division of Preventive Oncology, German Cancer Research Center (DKFZ) and National Center for Tumor Diseases (NCT), Heidelberg, Germany,

^145^Division of Cancer Prevention and Control, H. Lee Moffitt Cancer Center, 12902 Magnolia Dr., Tampa, Florida, USA,

^146^ Molecular Medicine Center and Department of Medical Chemistry and Biochemistry, Medical University - Sofia, 2 Zdrave St, 1431, Sofia, Bulgaria,

^147^ Australian Prostate Cancer Research Centre-Qld, Institute of Health and Biomedical Innovation and School of Biomedical Science, Queensland University of Technology, Brisbane, Australia,

^148^ Department of Genetics, Portuguese Oncology Institute, Porto, Portugal and Biomedical Sciences Institute (ICBAS), Porto University, Porto, Portugal,

^149^The University of Surrey, Guildford, Surrey, GU2 7XH, UK,

^150^German Cancer Consortium (DKTK), German Cancer Research Center (DKFZ), Heidelberg, Germany,

^151^Centre for Molecular Oncology, Barts Cancer Institute, Queen Mary University of London, John Vane Science Centre, Charterhouse Square, London, EC1M 6BQ, UK

**COGS acknowledgement and funding:**

This study would not have been possible without the contributions of the following: Per Hall (COGS); Douglas F. Easton, Paul Pharoah, Kyriaki Michailidou, Manjeet K. Bolla, Qin Wang (BCAC), Andrew Berchuck (OCAC), Rosalind A. Eeles, Douglas F. Easton, Ali Amin Al Olama, Zsofia Kote-Jarai, Sara Benlloch (PRACTICAL), Georgia Chenevix-Trench, Antonis Antoniou, Lesley McGuffog, Fergus Couch and Ken Offit (CIMBA), Joe Dennis, Alison M. Dunning, Andrew Lee, and Ed Dicks, Craig Luccarini and the staff of the Centre for Genetic Epidemiology Laboratory, Javier Benitez, Anna Gonzalez-Neira and the staff of the CNIO genotyping unit, Jacques Simard and Daniel C. Tessier, Francois Bacot, Daniel Vincent, Sylvie LaBoissière and Frederic Robidoux and the staff of the McGill University and Génome Québec Innovation Centre, Stig E. Bojesen, Sune F. Nielsen, Borge G. Nordestgaard, and the staff of the Copenhagen DNA laboratory, and Julie M. Cunningham, Sharon A. Windebank, Christopher A. Hilker, Jeffrey Meyer and the staff of Mayo Clinic Genotyping Core Facility

Funding for the iCOGS infrastructure came from: the European Community's Seventh Framework Programme under grant agreement n° 223175 (HEALTH-F2-2009-223175) (COGS), Cancer Research UK (C1287/A10118, C1287/A 10710, C12292/A11174, C1281/A12014, C5047/A8384, C5047/A15007, C5047/A10692, C8197/A16565), the National Institutes of Health (CA128978) and Post-Cancer GWAS initiative (1U19 CA148537, 1U19 CA148065 and 1U19 CA148112 - the GAME-ON initiative), the Department of Defence (W81XWH-10-1-0341), the Canadian Institutes of Health Research (CIHR) for the CIHR Team in Familial Risks of Breast Cancer, Komen Foundation for the Cure, the Breast Cancer Research Foundation, and the Ovarian Cancer Research Fund.

**Transdisciplinary Research in Cancer of the Lung (TRICL) Research Team**:

[Hung RJ](http://www.ncbi.nlm.nih.gov/pubmed/?term=Hung RJ%5BAuthor%5D&cauthor=true&cauthor_uid=26905588)^152^, [Han Y](http://www.ncbi.nlm.nih.gov/pubmed/?term=Han Y%5BAuthor%5D&cauthor=true&cauthor_uid=26905588)^153^, [Brennan P](http://www.ncbi.nlm.nih.gov/pubmed/?term=Brennan P%5BAuthor%5D&cauthor=true&cauthor_uid=26905588)^154^, [Bickeböller H](http://www.ncbi.nlm.nih.gov/pubmed/?term=Bickeböller H%5BAuthor%5D&cauthor=true&cauthor_uid=26905588)^155^, [Rosenberger A](http://www.ncbi.nlm.nih.gov/pubmed/?term=Rosenberger A%5BAuthor%5D&cauthor=true&cauthor_uid=26905588)^155^, [Houlston RS](http://www.ncbi.nlm.nih.gov/pubmed/?term=Houlston RS%5BAuthor%5D&cauthor=true&cauthor_uid=26905588)^156^, [Caporaso N](http://www.ncbi.nlm.nih.gov/pubmed/?term=Caporaso N%5BAuthor%5D&cauthor=true&cauthor_uid=26905588)^157^, [Landi MT](http://www.ncbi.nlm.nih.gov/pubmed/?term=Landi MT%5BAuthor%5D&cauthor=true&cauthor_uid=26905588)^157^, [Heinrich J](http://www.ncbi.nlm.nih.gov/pubmed/?term=Heinrich J%5BAuthor%5D&cauthor=true&cauthor_uid=26905588)^158^, [Risch A](http://www.ncbi.nlm.nih.gov/pubmed/?term=Risch A%5BAuthor%5D&cauthor=true&cauthor_uid=26905588)^159^, [Wu X](http://www.ncbi.nlm.nih.gov/pubmed/?term=Wu X%5BAuthor%5D&cauthor=true&cauthor_uid=26905588)^160^, [Ye Y](http://www.ncbi.nlm.nih.gov/pubmed/?term=Ye Y%5BAuthor%5D&cauthor=true&cauthor_uid=26905588)^160^, [Christiani DC](http://www.ncbi.nlm.nih.gov/pubmed/?term=Christiani DC%5BAuthor%5D&cauthor=true&cauthor_uid=26905588)^161,162^, [Amos CI](http://www.ncbi.nlm.nih.gov/pubmed/?term=Amos CI%5BAuthor%5D&cauthor=true&cauthor_uid=26905588)^153^

^152^Lunenfeld-Tanenbaum Research Institute of Mount Sinai Hospital, Toronto, Ontario, Canada ^153^Department of Biomedical Data Science, Geisel School of Medicine, Dartmouth College, Hanover, NH  ^154^Genetic Epidemiology Group, International Agency for Research on Cancer (IARC), Lyon, France ^155^Department of Genetic Epidemiology, University Medical Center, Georg-August-University Göttingen, Göttingen, Germany ^156^Division of Genetics and Epidemiology, The Institute of Cancer Research, London, United Kingdom ^157^Division of Cancer Epidemiology and Genetics, National Cancer,Institute, National Institutes of Health, Bethesda, MD, USA ^158^Helmholtz Centre Munich, German Research Centre for Environmental Health, Institute of Epidemiology I, Neuherberg, Germany ^159^Department of Molecular Biology,University of Salzburg, Salzburg, Austria ^160^Department of Epidemiology, UT MD Anderson Cancer Center, Houston, TX ^161^Massachusetts General Hospital, Boston, Massachusetts ^162^Department of Environmental Health, Harvard School of Public Health, Boston, Massachusetts

**Supplementary Figures:**

**Supplementary Figure 1: Stratified Q-Q plots for schizophrenia (SCZ) given other cancer types**


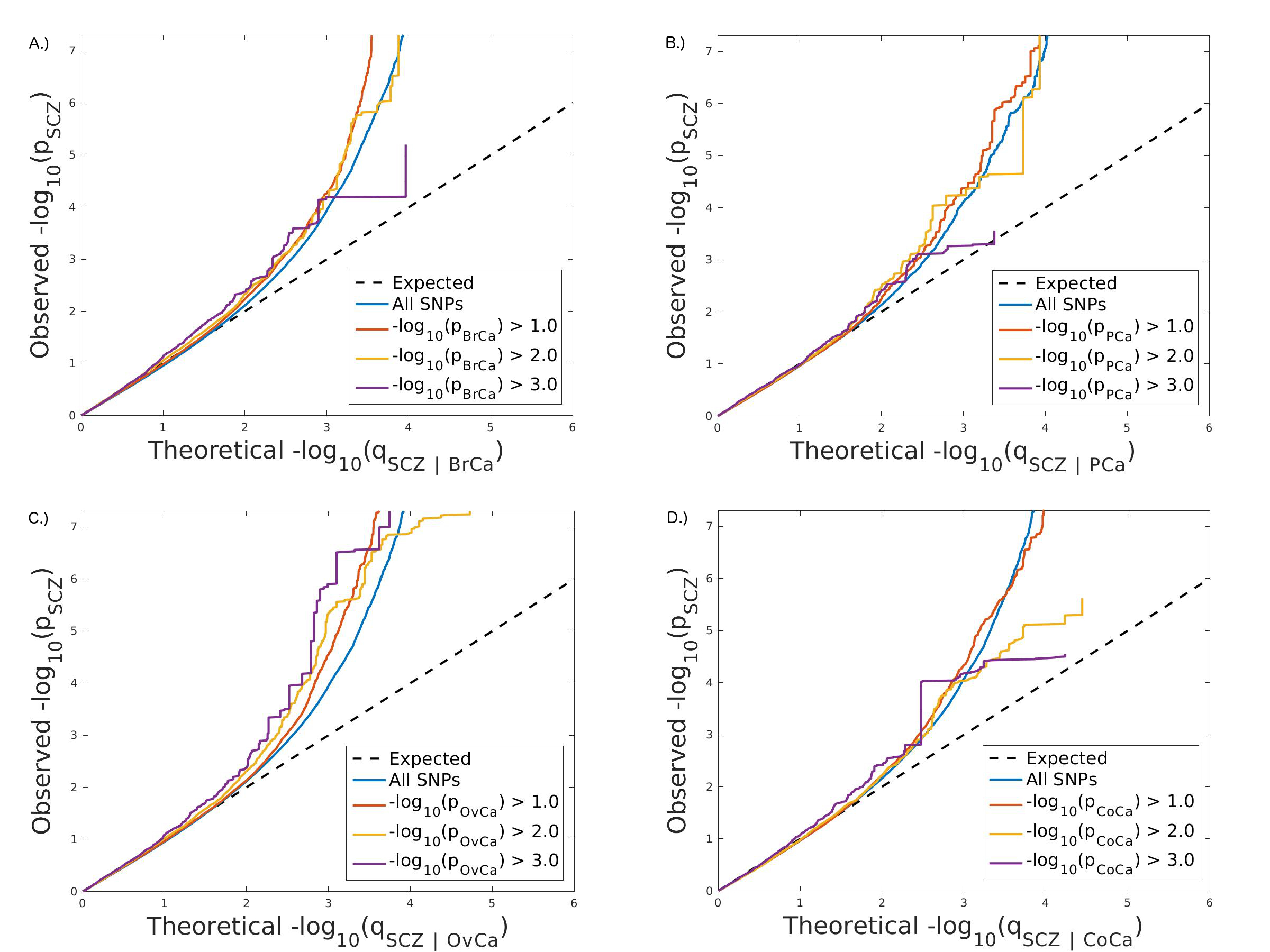


Supplementary Figure 1: Stratified Q-Q plots of theoretical vs empirical -log_10_ *p*-values (corrected for genomic control) in schizophrenia (SCZ) below the standard GWAS threshold of -log10 p-values equal to 7.3 (equals *p*-values above 5 x 10^-8^) as a function of significance of association with A.) breast cancer (BrCa), B.) prostate cancer (PCa), C.) ovarian cancer (OvCa), D.) and colon cancer (CoCa) at the level of p < 1 (all SNPs), p <0.1, p < 0.01, p < 0.001 respectively. Dotted lines indicate the theoretical line in case of no association. Variants mapping to the major histocompatibility complex (MHC) haven been removed prior to the analysis.

**Supplementary Figure 2: Stratified Q-Q plot for schizophrenia (SCZ) given lung cancer (LgCa) including single nucleotide polymorphisms (SNPs) in the major histocompatibility complex (MHC).**

**
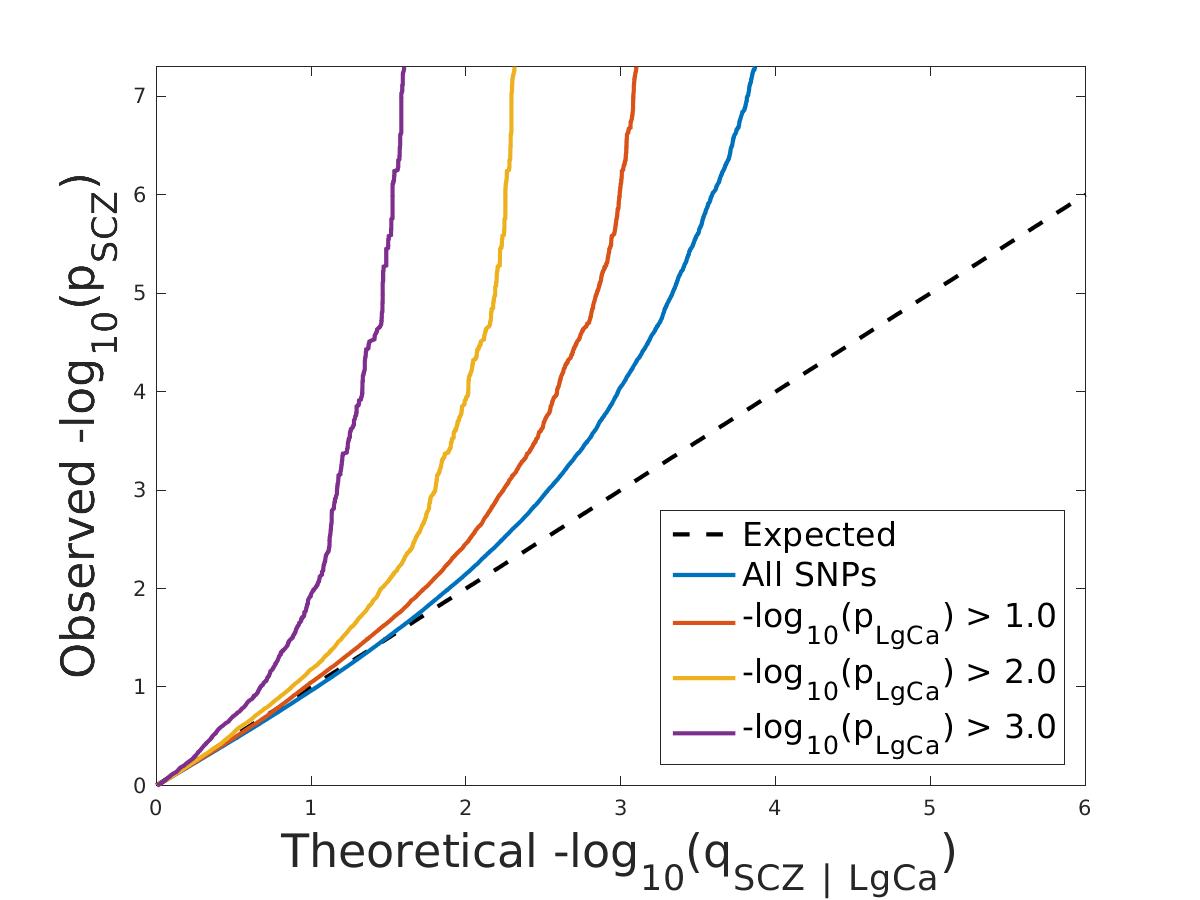
**

Supplementary Figure 2: Stratified Q-Q plots of theoretical vs empirical -log_10_ *p*-values (corrected for genomic control) in schizophrenia (SCZ) below the standard GWAS threshold of -log10 p-values equal to 7.3 (equals *p*-values above 5 x 10^-8^) as a function of significance of association with lung cancer (LgCa) at the level of p < 1 (all SNPs), p <0.1, p < 0.01, p < 0.001 respectively. Dotted lines indicate the theoretical line in case of no association. Genetic variants mapping to MHC have been included to the analysis. Please see Main Figure 1 for the analysis excluding the MHC region.

**Supplementary Figure 3: Stratified Q-Q plot for schizophrenia (SCZ) given lung cancer, and for lung cancer given schizophrenia (SCZ)**


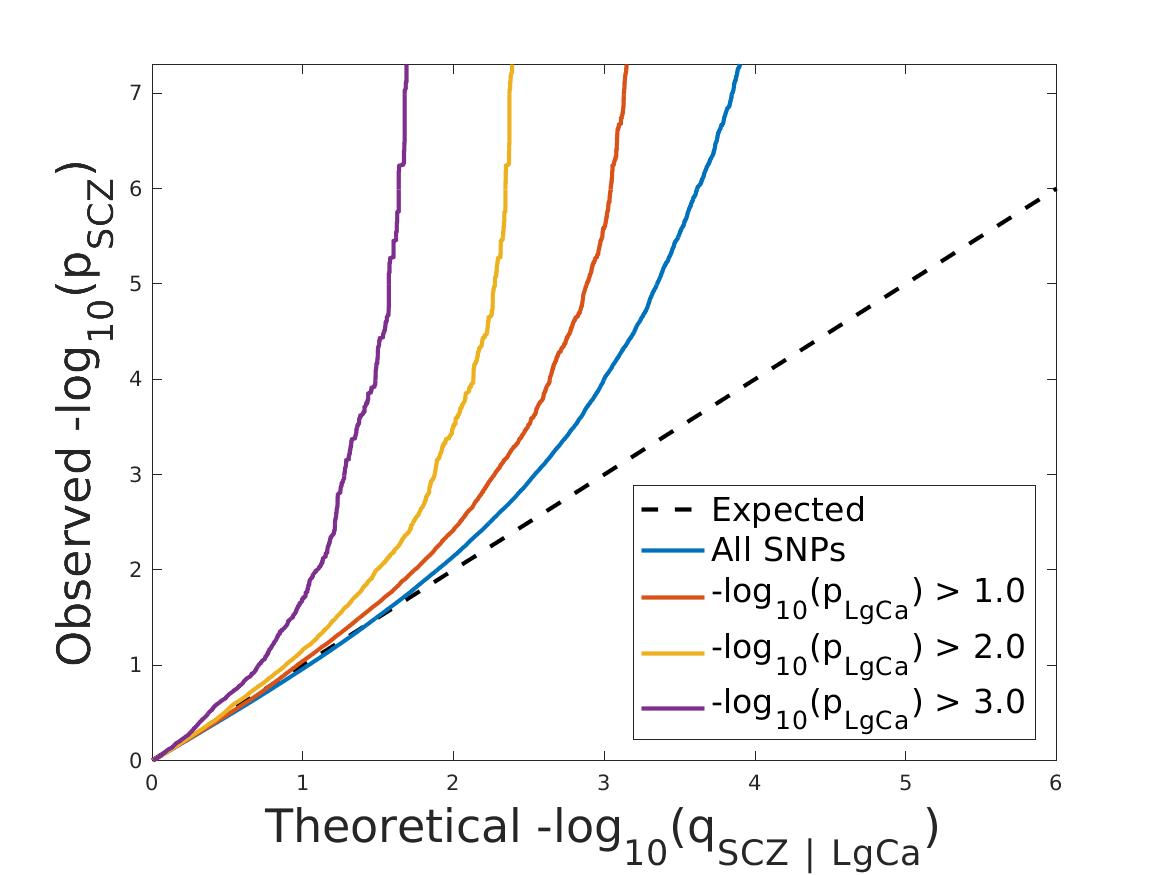

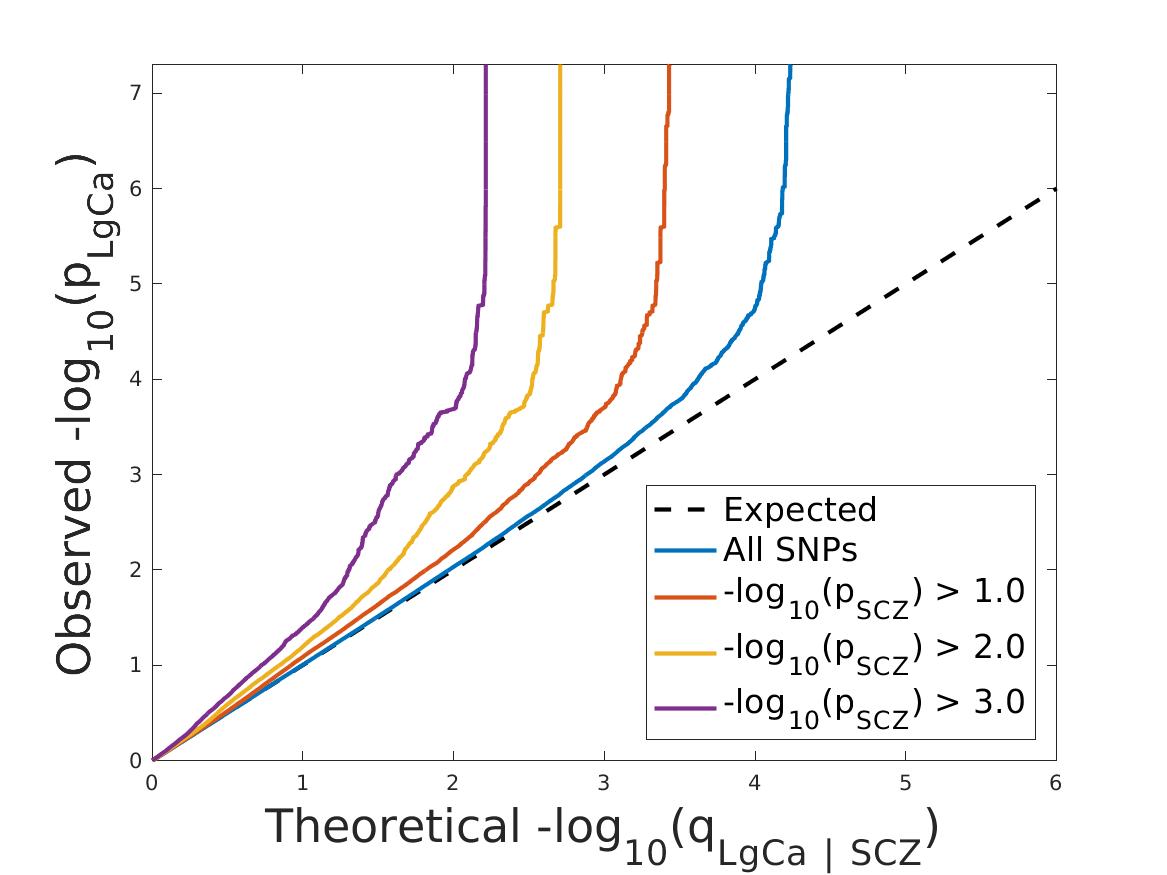


A.)

B.)

Supplementary Figure 3**:** In order to demonstrate the symmetry of enrichment we present alongside stratified Q-Q plots for A.) schizophrenia (SCZ) given lung cancer (LgCa) as shown in Main Figure 1 and B.) lung cancer (LgCa) given schizophrenia (SCZ). The MHC region has been excluded prior to the analysis.

**Supplementary Figure 4: Functional evidence for rs2081361 (11q12)**


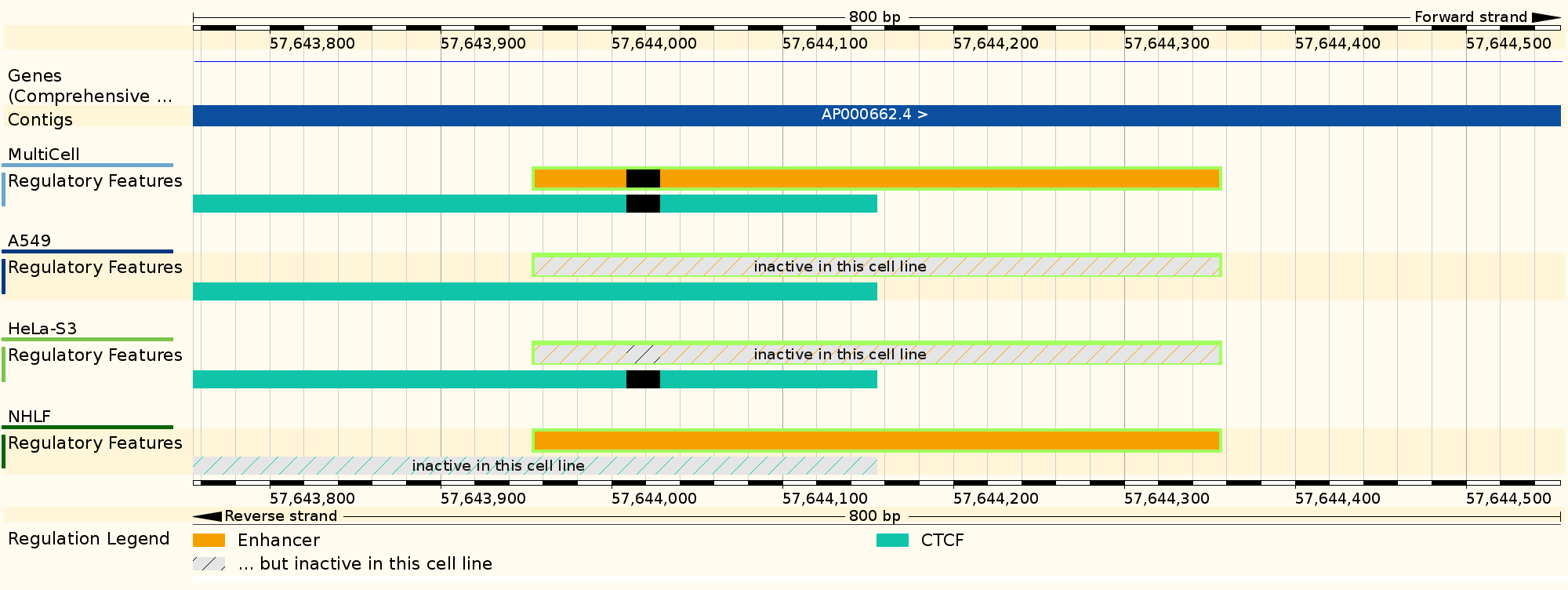


Supplementary Figure 4: Functional evidence in lung cell lines (A549 and NHLF) and HeLa cells as retrieved from ENSEMBL for rs2081361 the lead variant of the 11q12 locus.

**Supplementary Figure 5: Stratified Q-Q plot for schizophrenia (SCZ) given cigarettes per day (CPD) before and after excluding the 15q25 locus**


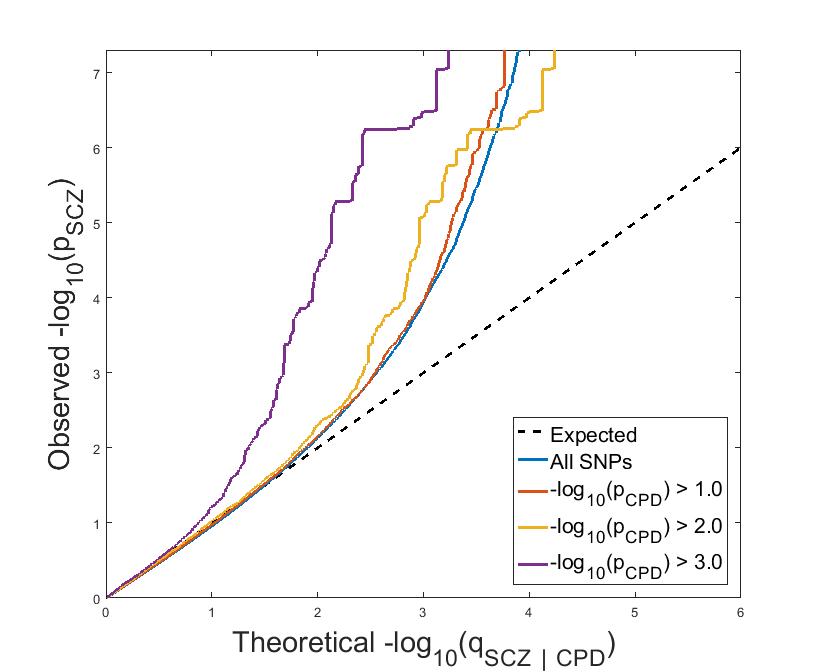


A.)


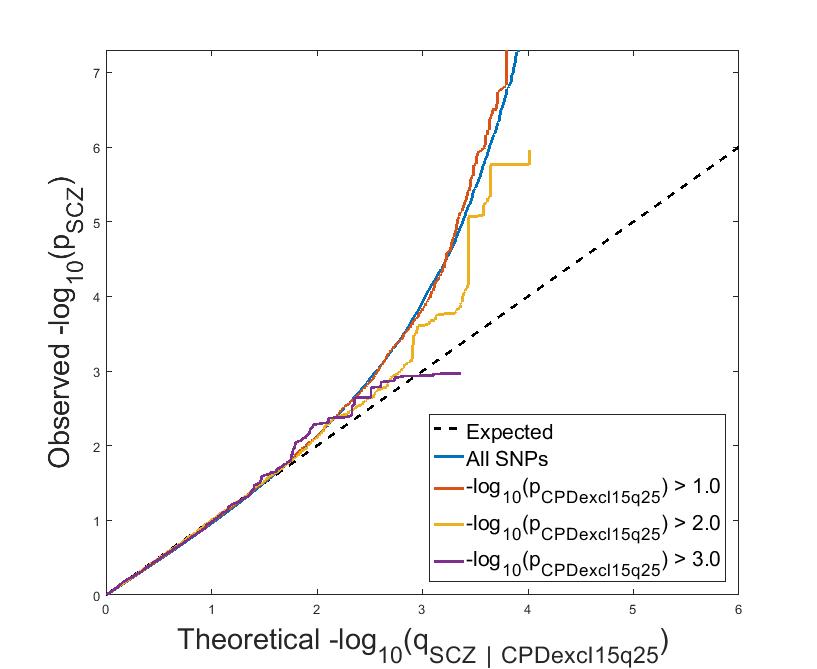


B.)

Supplementary Figure 5 A.) Stratified Q-Q plot for schizophrenia (SCZ) given cigarettes per day (CPD) after removing the major histocompatibility complex (MHC, genomic position (hg 19): chr6:29528318- 33373649). B.) Stratified Q-Q plot for schizophrenia (SCZ) given cigarettes per day (CPD) after removing the major histocompatibility complex (MHC, genomic position (hg 19): chr6:29528318-33373649) and additionally genes mapping to the nicotinic acetylcholine receptors (genomic position (hg 19) chr15: 78,686,690-79,231,478).

**Supplementary Figure 6: Stratified Q-Q plot for schizophrenia (SCZ) given subtypes of lung cancer**


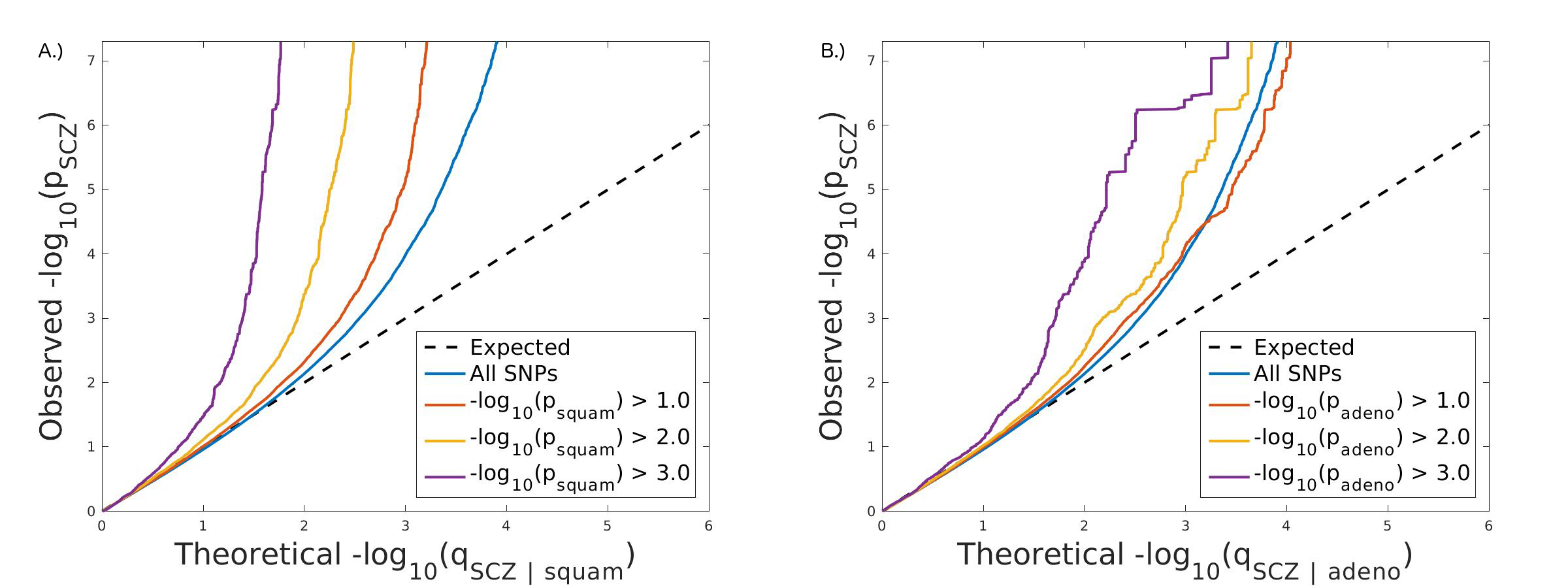


Supplementary Figure 6: Stratified Q-Q plot for schizophrenia (SCZ) given A.) squamous cell carcinoma (SQUAM) and B.) adenocarcinoma (ADENO) of the lung after removing the major histocompatibility complex (MHC).

**Supplementary Figure 7: Stratified Q-Q plot for schizophrenia (SCZ) given lung cancer using differing parameters for pruning**


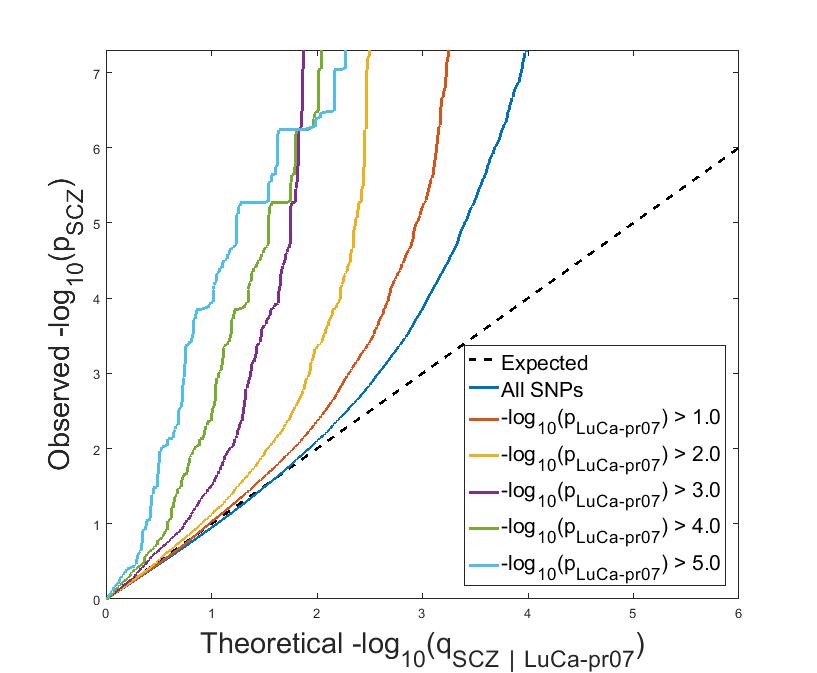


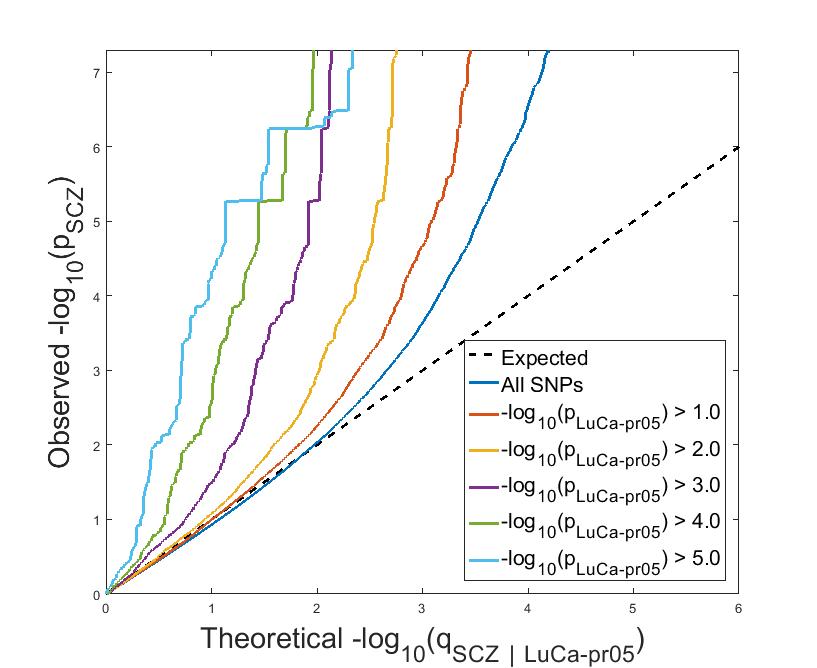


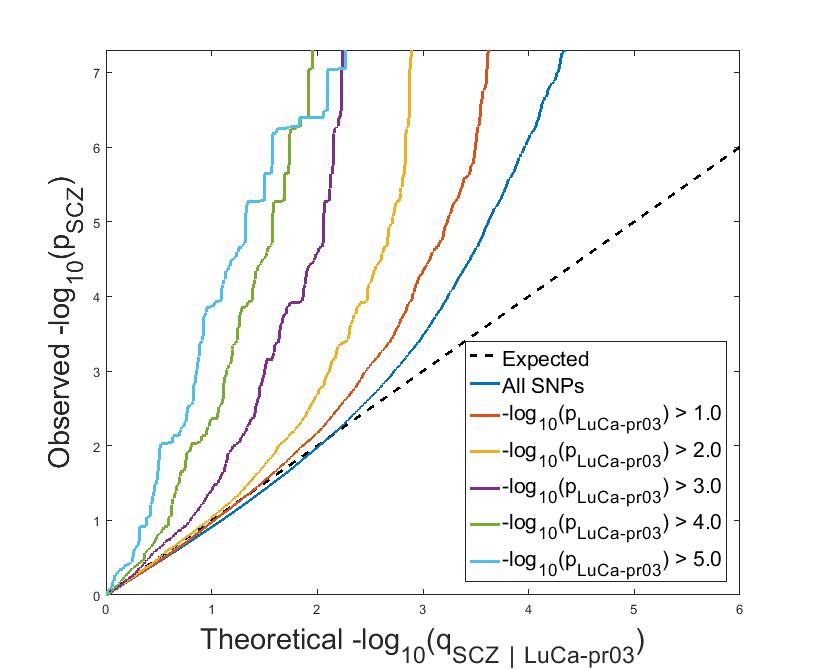


Supplementary Figure 7: In order to demonstrate the impact of pruning we present here stratified Q-Q plots for schizophrenia (SCZ) given lung cancer (LgCa) when using differing LD parameters (*r^2^* of *0.7* top panel, *0.5* middle panel, and *0.3* bottom panel) for pruning. Stratified Q-Q plots of theoretical vs empirical -log_10_ *p*-values (corrected for genomic control) in SCZ below the standard GWAS threshold of -log10 p-values equal to 7.3 (equals *p*-values above 5 x 10^-8^) as a function of significance of association with LgCa at the level of p < 1 (all SNPs), p <0.1, p < 0.01, p < 0.001, p<0.0001, p<0.00001 respectively. Dotted lines indicate the theoretical line in case of no association. The MHC region has been excluded prior to the analysis.

**Supplementary Tables:**

**Supplementary Table 1: Sample description of the contributing GWAS studies.**

|  | **Cases** | **Controls** | **Reference** |
| --- | --- | --- | --- |
| SCZ | 32,405 | 42,221 | Schizophrenia Working Group of the Psychiatric Genomics, C., *Biological insights from 108 schizophrenia-associated genetic loci.* Nature, 2014. **511**(7510): p. 421-7. |
| Lung cancer  Adenocarcinoma Squamous cell carcinoma | 12,160  3,718  3,422 | 16,838  15,871  16,015 | Timofeeva, M.N., et al., *Influence of common genetic variation on lung cancer risk: meta-analysis of 14 900 cases and 29 485 controls.* Hum Mol Genet, 2012. **21**(22): p. 4980-95. |
| Breast cancer | 15,863 | 40,022 | Michailidou, K., et al., *Large-scale genotyping identifies 41 new loci associated with breast cancer risk.* Nat Genet, 2013. **45**(4): p. 353-61, 361e1-2. |
| Prostate cancer | 25,074 | 24,272 | Eeles, R.A., et al., *Identification of 23 new prostate cancer susceptibility loci using the iCOGS custom genotyping array.* Nat Genet, 2013. **45**(4): p. 385-91, 391e1-2. |
| Colon cancer | 5,100 | 7,529 | Schumacher FR, et al. Corrigendum: genome-wide association study of colorectal cancer identifies six new susceptibility loci. Nature communications 2015; 6: 8739. |
| Ovarian cancer | 3,995 | 3,277 | Pharoah, P.D., et al., *GWAS meta-analysis and replication identifies three new susceptibility loci for ovarian cancer.* Nat Genet, 2013. **45**(4): p. 362-70, 370e1-2. |
| Smoking habits:  Cigarettes per day (CPD), age of smoking initiation (LOGONSET), initiation (EVRSMK), cessation (FORMER) | 74,053 | | Tobacco, Genetics C*. Genome-wide meta-analyses identify multiple loci associated with smoking behavior*. *Nat Genet* 2010; **42**(5): 441-7 |

**Supplementary Table 2: Test for fold-enrichment of the strata in the Q-Q plots.**

Fold enrichment of genetic association with schizophrenia given the three Q-Q plot strata (-log10pval>1,2, and 3, or equivalently p <0.1, p < 0.01, p < 0.001) based on the secondary cancer trait. Prostate cancer was excluded due to the low coverage of the costumised SNP array. Final *p*-values are adjusted for multiple testing of four cancer traits and three strata each. LD-score regression was used to derive these parameters, SNPs in the MHC region were excluded prior to analysis.

|  | stratum | fold enrichment | enrichment_SE | enrichment_p | adjusted p-value |
| --- | --- | --- | --- | --- | --- |
| Breast cancer | -log10pval >1 | 1.331 | 0.158 | 0.035 | 0.415 |
|  | -log10pval >2 | 1.761 | 0.503 | 0.129 | 1.000 |
|  | -log10pval >3 | 1.884 | 1.194 | 0.459 | 1.000 |
| Colon cancer | -log10pval >1 | 0.968 | 0.181 | 0.861 | 1.000 |
|  | -log10pval >2 | 1.084 | 0.572 | 0.884 | 1.000 |
|  | -log10pval >3 | 4.505 | 3.152 | 0.267 | 1.000 |
| Ovarian cancer | -log10pval >1 | 1.168 | 0.158 | 0.289 | 1.000 |
|  | -log10pval >2 | 1.715 | 0.715 | 0.320 | 1.000 |
|  | -log10pval >3 | 4.436 | 11.665 | 0.769 | 1.000 |
| Lung cancer | -log10pval >1 | 1.424 | 0.138 | 0.002 | **0.024** |
|  | -log10pval >2 | 2.190 | 0.597 | 0.049 | 0.591 |
|  | -log10pval >3 | 6.512 | 3.043 | 0.072 | 0.860 |

**Supplementary Table 3A: eQTL lookup of the lead SNPs and LD proxies from GTEx in lung and brain tissue**

The following data is taken from the GTEx project portal (V6p). The table includes information on the three lead SNPs of the cross-phenotype associated loci (Main Table 1), its LD proxies and the respective LD (r^2^), furthermore we include reference and alternative allele of the LD proxy. For the eQTL association we report tissue, gene, *p*-value and effect size. The effect size is computed as the effect of the alternative allele (ALT) relative to the reference allele (REF) in the human genome reference GRCh37/hg19.

|  | lead SNPs | location | LD proxy | LD | ref | alt | tissue | gene | p-value | effect size | LD proxy | LD | tissue | gene | p-value | effect size |
| --- | --- | --- | --- | --- | --- | --- | --- | --- | --- | --- | --- | --- | --- | --- | --- | --- |
| 1 | rs7749305 | 6p22.1 | rs28360634 | 1 | T | C | lung | *BTN3A2* | 1.60E-23 | -0.95 | rs28360634 | 1 | brain (frontal cortex) | *BTN3A2* | 1.00E-07 | -1 |
|  |  |  | rs72839477 | 1 | C | T | lung | *BTN3A2* | 1.30E-27 | -1.1 | rs72839477 | 1 | brain (frontal cortex) | *BTN3A2* | 1.00E-07 | -1 |
| 2 | rs2081361 | 11q12.1 | rs2081361 | 1 | C | T | lung | *TIMM10* | 0.0000013 | -0.25 | rs2081361 | 1 | brain (cerebellar hemisphere) | *LRRC55* | 0.000083 | 0.55 |
| 3 | rs8042374 | 15q25.1 | rs2904130 | 0.89 | C | G | lung | *CHRNA5* | 4.30E-08 | -0.42 | rs2904130 | 0.89 | brain (caudate) | *CHRNA5* | 0.000011 | -0.61 |
|  |  |  | rs2904130 | 0.89 | C | *G* | brain (caudate) | *CHRNA3* | 1.10E-23 | -0.96 | rs2904130 | 0.89 | brain (nucleus accumbens) | *CHRNA3* | 6.80E-17 | -0.96 |

**Supplementary Table 3B: eQTL lookup of the lead SNPs in the BRAINEAC**

The BRAINEAC resource reports eQTL data for ten different brain tissues, the minimum *p*-value (min_*p-*value) and a *p*-value over the averaged expression of all tissues (aveALL). Abbreviations for further brain tissues are: CRBL, cerebellum; FCTX, frontal cortex; HIPP, hippocampus; MEDU, medulla oblongata; OCTX, occipital cortex; PUTM, putamen; SNIG, substantia nigra; TCTX, temporal cortex; THAL, thalamus, WHMT, white matter. tID is the transcript cluster ID from Affymetrix Human Exon 1.0 ST. We report the three top transcripts per gene.

| **rs28360634** | **6p22.1** |  |  |  |  |  |  |  |  |  |  |  |  |  |  |  |
| --- | --- | --- | --- | --- | --- | --- | --- | --- | --- | --- | --- | --- | --- | --- | --- | --- |
| **Symbol** | **exprID** | **chr** | **start** | **stop** | **aveALL** | **CRBL** | **FCTX** | **HIPP** | **MEDU** | **OCTX** | **PUTM** | **SNIG** | **TCTX** | **THAL** | **WHMT** | **min_p-value** |
| BTN3A2 | t2899333 | chr6 | 26376757 | 26378535 | 5.80E-14 | 4.30E-07 | 1.40E-04 | 2.50E-06 | 2.10E-03 | 3.60E-02 | 3.60E-04 | 8.60E-07 | 7.10E-06 | 3.00E-07 | 9.60E-04 | 5.80E-14 |
| BTN3A2 | 2899339 | chr6 | 26376757 | 26378535 | 2.30E-11 | 8.90E-03 | 8.10E-03 | 4.90E-03 | 4.40E-03 | 1.50E-02 | 3.70E-03 | 1.00E-04 | 1.20E-01 | 1.10E-03 | 7.20E-03 | 2.30E-11 |
| BTN3A2 | 2899336 | chr6 | 26376757 | 26378535 | 3.00E-11 | 8.00E-04 | 8.40E-05 | 2.20E-05 | 2.30E-02 | 3.10E-03 | 5.10E-04 | 1.70E-05 | 2.40E-07 | 6.70E-07 | 1.10E-02 | 3.00E-11 |
| **rs2081361** | **11q12.1** |  |  |  |  |  |  |  |  |  |  |  |  |  |  |  |
| **Symbol** | **exprID** | **chr** | **start** | **stop** | **aveALL** | **CRBL** | **FCTX** | **HIPP** | **MEDU** | **OCTX** | **PUTM** | **SNIG** | **TCTX** | **THAL** | **WHMT** | **min_p-value** |
| TIMM10 | 3373952 | chr11 | 57295938 | 57308980 | 8.10E-04 | 6.50E-01 | 2.30E-01 | 4.40E-03 | 2.50E-02 | 8.40E-02 | 4.30E-04 | 3.70E-01 | 1.90E-01 | 3.00E-02 | 6.20E-02 | 4.30E-04 |
| TIMM10 | 3373951 | chr11 | 57295938 | 57308980 | 7.00E-03 | 3.90E-01 | 6.90E-02 | 4.30E-01 | 1.60E-01 | 4.60E-01 | 7.00E-04 | 3.40E-01 | 6.80E-01 | 6.60E-02 | 4.20E-02 | 7.00E-04 |
| TIMM10 | 3373953 | chr11 | 57295938 | 57308980 | 9.80E-03 | 9.10E-01 | 1.10E-01 | 4.70E-01 | 9.00E-02 | 5.60E-01 | 5.40E-02 | 2.40E-02 | 6.80E-02 | 1.20E-02 | 3.70E-02 | 9.80E-03 |
| LRRC55 | 3331180 | chr11 | 56801304 | 56959187 | 6.00E-01 | 5.30E-03 | 2.10E-01 | 2.00E-01 | 6.40E-01 | 1.90E-01 | 8.00E-01 | 7.00E-01 | 3.10E-01 | 7.60E-02 | 8.80E-01 | 5.30E-03 |
| LRRC55 | 3331174 | chr11 | 56801304 | 56959187 | 7.50E-01 | 2.70E-01 | 5.20E-01 | 6.60E-01 | 3.60E-01 | 6.80E-03 | 4.80E-01 | 7.10E-01 | 6.20E-02 | 7.50E-01 | 7.00E-01 | 6.80E-03 |
| LRRC55 | 3331177 | chr11 | 56801304 | 56959187 | 3.70E-01 | 4.00E-01 | 9.70E-01 | 7.00E-01 | 7.60E-01 | 8.50E-02 | 8.60E-01 | 8.70E-01 | 3.20E-02 | 2.90E-01 | 2.20E-01 | 3.20E-02 |
| **rs2904130** | **15q25.1** |  |  |  |  |  |  |  |  |  |  |  |  |  |  |  |
| **Symbol** | **exprID** | **chr** | **start** | **stop** | **aveALL** | **CRBL** | **FCTX** | **HIPP** | **MEDU** | **OCTX** | **PUTM** | **SNIG** | **TCTX** | **THAL** | **WHMT** | **min_p-value** |
| CHRNA5 | 3603447 | chr15 | 78857902 | 78886460 | 6.10E-12 | 1.90E-02 | 4.50E-07 | 6.20E-06 | 7.90E-02 | 8.50E-06 | 6.20E-04 | 8.00E-06 | 2.60E-06 | 1.50E-02 | 3.70E-04 | 6.10E-12 |
| CHRNA5 | t3603436 | chr15 | 78857902 | 78886460 | 1.60E-11 | 6.40E-03 | 1.00E-09 | 7.10E-06 | 6.10E-02 | 1.10E-05 | 1.20E-04 | 5.40E-08 | 2.60E-06 | 7.70E-03 | 2.50E-04 | 1.60E-11 |
| CHRNA5 | 3603446 | chr15 | 78857902 | 78886460 | 3.90E-11 | 2.60E-04 | 1.00E-06 | 1.10E-03 | 9.10E-02 | 1.00E-03 | 5.50E-04 | 9.60E-08 | 1.80E-04 | 3.10E-02 | 3.70E-03 | 3.90E-11 |

**Supplementary Table 4: Independent (r^2^ < 0.2), cross–phenotype associated loci for schizophrenia (SCZ) and lung cancer including information of smoking traits**

Independent (*r^2^ <0.2*) loci associated with both schizophrenia (SCZ) and lung cancer (LgCa) as defined by conjunction false discovery rates (ConjFDR < 0.01), as presented in Main Table 1. In addition, we include more information on smoking traits including cigarettes per day (CPD), smoking initiation (EVRSMK), smoking cessation (FORMER), and age of smoking initiation (ONSET).

|  | **lead SNP** | **Gene** | **Band** | **A1** | **A2** | ***p-*value**  **SCZ** | ***p-*value**  **LgCa** | ***p-*value**  **CPD** | ***p-*value**  **EVRSMK** | ***p-*value**  **FORMER** | ***p-*value**  **ONSET** | ***z-*score**  **SCZ** | ***z-s*core**  **LgCa** | ***z*-score**  **CPD** | ***z-*score**  **EVRSMK** | ***z-s*core**  **FORMER** | ***z-s*core**  **ONSET** | **ConjFDR**  **SCZ_LgCa** |
| --- | --- | --- | --- | --- | --- | --- | --- | --- | --- | --- | --- | --- | --- | --- | --- | --- | --- | --- |
| 1 | rs7749305 | ZNF184 | 6p22.1 | T | C | 2.39E-17 | 5.08E-06 | NaN | NaN | NaN | NaN | 8.47 | -4.56 | NaN | NaN | NaN | NaN | 2.34E-04 |
| 2 | rs2081361 | AK096335 | 11q12.1 | C | T | 2.53E-04 | 1.67E-05 | 2.52E-01 | 7.18E-01 | 2.93E-01 | 7.96E-02 | -3.66 | -4.31 | -1.15 | -0.36 | 1.05 | -1.75 | 5.88E-03 |
| 3 | rs8042374 | CHRNA3 | 15q25.1 | A | G | 2.06E-08 | 5.30E-32 | 5.09E-23 | 8.10E-01 | 8.41E-04 | 4.22E-01 | 5.61 | 11.77 | 9.88 | 0.24 | -3.34 | -0.80 | 9.25E-07 |

**Supplementary Table 5: Independent (r^2^ < 0.2), cross–phenotype associated loci for schizophrenia (SCZ) and squamous cell carcinoma (SQUAM) and adenocarcinoma (ADENO) of the lung.**

Cross-phenotype associations as defined by joint conjunction false discovery rates (conjfdr) < 0.01 between schizophrenia (SCZ) and squamous cell carcinoma (SQUAM) and adenocarcinoma (ADENO) of the lung. In addition, we include information on association with different smoking traits including cigarettes per day (CPD), smoking initiation (EVRSMK), smoking cessation (FORMER), and age of smoking initiation (ONSET). For each locus we report the lead SNP, closest annotated gene (gene), genomic position (Band), *p*-values (pval) and *z*-scores (zscore) for the specific traits. The major histocompatibility complex (MHC) was excluded in the analyses.

|  | **lead**  **SNP** | **Gene** | **Band** | **A1** | **A2** | ***p-*value SCZ** | ***p-*value SQUAM** | ***p-*value ADENO** | ***p-*value CPD** | ***p-*value EVRSMK** | ***p-*value FORMER** | ***p-*value ONSET** | ***z-*score SCZ** | ***z-s*core SQUAM** | ***z-s*core ADENO** | ***z*-score CPD** | ***z-*score EVRSMK** | ***z-s*core FORMER** | ***z-s*core ONSET** |
| --- | --- | --- | --- | --- | --- | --- | --- | --- | --- | --- | --- | --- | --- | --- | --- | --- | --- | --- | --- |
| 1 | rs13191445 | HIST1H1A | 6p22.2 | G | A | 2.75E-13 | 1.15E-04 | 4.53E-01 | 5.97E-02 | 3.57E-01 | 3.78E-01 | 4.85E-01 | 7.31 | -3.86 | -0.75 | -1.88 | -0.92 | 0.88 | -0.70 |
| 2 | rs7749305 | ZNF184 | 6p22.1 | T | C | 2.39E-17 | 2.45E-05 | 2.47E-01 | NaN | NaN | NaN | NaN | 8.47 | -4.22 | -1.16 | NaN | NaN | NaN | NaN |
| 3 | rs8042374 | CHRNA3 | 15q25.1 | A | G | 2.06E-08 | 4.32E-15 | 1.65E-11 | 5.09E-23 | 8.10E-01 | 8.41E-04 | 4.22E-01 | 5.61 | 7.85 | 6.73 | 9.88 | 0.24 | -3.34 | -0.80 |

|  | **lead**  **SNP** | **Gene** | **Band** | **A1** | **A2** | **ConjFDR SCZ_**  **SQUAM** | **ConjFDR SCZ_**  **ADENO** |
| --- | --- | --- | --- | --- | --- | --- | --- |
| 1 | rs13191445 | HIST1H1A | 6p22.2 | G | A | 7.87E-03 | 9.02E-01 |
| 2 | rs7749305 | ZNF184 | 6p22.1 | T | C | 1.87E-03 | 8.65E-01 |
| 3 | rs8042374 | CHRNA3 | 15q25.1 | A | G | 1.14E-06 | 1.42E-05 |
